# Longitudinal assessment of kidney function in migrant farm workers

**DOI:** 10.1016/j.envres.2021.111686

**Published:** 2021-07-14

**Authors:** Nicolás López-Gálvez, Rietta Wagoner, Robert A. Canales, Kacey Ernst, Jefferey L. Burgess, Jill de Zapien, Cecilia Rosales, Paloma Beamer

**Affiliations:** a San Diego State University Research Foundation, San Diego State University, 5250 Campanile Dr, San Diego, CA, 92182, USA; b Mel and Enid Zuckerman College of Public Health, University of Arizona, 1295 N. Martin Ave, PO 245210, Tucson, AZ, 85724, USA; c Interdisciplinary Program in Applied Mathematics, University of Arizona, 617 N. Santa Rita Ave, PO Box 210089, Tucson, AZ, 85721, USA

**Keywords:** Kidney function, Migrant farm workers, Heat stress, Pesticide exposure, Dehydration

## Abstract

Chronic kidney disease of unknown etiology (CKDu) is an epidemic that affects young agricultural workers in several warm regions of the world. However, there is a lack of monitoring of kidney issues in regions with extremely warm environments such as the Northwest of Mexico, a semi-arid region with a growing agricultural industry, where migrant and seasonal farm workers (MSFWs) travel to work in the fields. The objective of this study was to longitudinally assess kidney functioning of MSFWs in relation to pesticide exposure, heat stress and dehydration in a large-scale farm in Mexico. We enrolled 101 MSFWs, of whom 50 were randomly selected to work in an organic certified area and 51 were randomly selected to work in a conventional area. We also enrolled 50 office workers within the same region as a reference group. We collected urine and blood samples from all workers in addition to demographic, behavioral, and occupational characteristics. The physiological strain index (PSI) was used to estimate workers’ heat strain. Sampling was conducted at pre-harvest (March) and late in the harvest (July). Linear mixed models were built with the estimated glomerular filtration rate (eGFR) as the dependent variable. We found a significant decrease in kidney function in MSFWs compared to office workers. By the late harvest, one MSFW developed kidney disease, two MSFWs suffered a kidney injury, and 14 MSFWs were at risk of a kidney injury. We found that the eGFR in MSFWs decreased significantly from pre-harvest (125 ± 13.0 mL/min/1.73 m^2^) to late harvest (109 ± 13.6 mL/min/1.73 m^2^) (p < 0.001), while no significant change was observed in office workers. The eGFR was significantly lower in MSFWs who worked in the conventional field (101.2 ± 19.4 mL/min/1.73 m^2^) vs the organic field (110.9 ± 13.6 mL/min/1.73 m^2^) (p = 0.002). In our final model, we found that dehydration was associated with the decrease of eGFR. We also found an interaction between heat strain and job category, as a significant decline in eGFR by job category (conventional/organic MSFWs and office workers) was related to an increase in heat strain. This suggests that pesticide exposure needs to be considered in combination with heat stress and dehydration. This study provides valuable information on kidney function in MSFWs, and it shows the importance of early long-term monitoring in farm workers in other regions where CKDu has not been evaluated yet.

## 1. Introduction

The prevalence of chronic kidney disease (CKD) is rising worldwide and has become a global health problem, as it is one of the fastest growing causes of mortality globally (Levin et al., 2017; Webster et al., 2017). People with kidney disease have difficulties filtering their blood, which produces a buildup of waste in the body and can lead to other chronic health issues such as cardiovascular disease, mineral bone disease and anemia (Webster et al., 2017). A subtype of CKD, chronic kidney disease of undetermined cause (CKDu), has gained significant attention in recent years because its cause is yet to be determined and it occurs in younger people without any traditional risk factors associated with CKD such as age, diabetes, hypertension, obesity, lipoprotein levels and tobacco usage (Laws et al., 2016; Ramirez-Rubio et al., 2013; Wesseling et al., 2014; Wijkström et al., 2013). Several epidemiological studies have reported a growing global epidemic of CKDu, with the prevalence steadily rising among vulnerable populations in Sri Lanka, Egypt, India, in the coastal zones of El Salvador, Nicaragua, Costa Rica and Southern Mexico (Brooks et al., 2012; Correa-Rotter et al., 2014; El-Arbagy et al., 2016; Jayasekara et al., 2015; Laws et al., 2016; Rajapurkar et al., 2012; Ramirez-Rubio et al., 2013; Wesseling et al., 2015).

CKDu disproportionately affects men in their 20s–50s who work in agriculture (Correa-Rotter et al., 2014). While the risk factors of CKDu are not fully understood, several factors have been proposed to explain the pattern of this disease, including: exposure to pesticides, pain medications, heavy metals, and heat stress (Correa-Rotter et al., 2014; Laws, 2015; Peraza et al., 2012; Ramirez-Rubio et al., 2013; Valcke et al., 2017; Wesseling et al., 2014). However, most of the existing published research on the topic has suggested that repeated exposure to high heat levels in combination with strenuous labor and dehydration may be the main risk factor associated with the development of CKDu (Crowe et al., 2013; Glaser et al., 2020; Hansson et al. 2019, 2020; Johnson et al., 2014; Peraza et al., 2012; Wesseling et al. 2016, 2020). Within the next few decades, the number of hot days (over 30 °C) and heat waves are expected to rise globally due to climate change; heat-related injuries and illnesses, such as kidney damage, are also expected to increase, which in part has led to the International Society of Nephrology recognizing CKDu as a global research priority (Levin et al., 2017; Xiang et al., 2014). Several studies focused on CKDu have found that workers in other industries such as construction, brickmaking, and mining also appear to be affected with kidney problems associated with high working temperatures and heavy manual labor (Gallo-Ruiz et al., 2019; Scammell et al., 2019). Although it has been proposed that heat-stress nephropathy may be the main factor related to CKDu, due to repeated dehydration and exposure to occupational heat stress, it remains unproven and it is likely that the causal mechanism of CKDu is a multifactorial one that includes several occupational risk factors that should be evaluated (Laws, 2015; Ramirez-Rubio et al., 2013; Scammell et al., 2019). Thus, a diverse combination of factors including pesticide exposure, pain medications, heat stress, and heavy metals must be considered (Nerbass et al., 2017).

CKDu is usually detected at a very late stage because there are no evident early clinical symptoms and there is a lack of surveillance studies and infrastructure to help monitor this disease, particularly among these younger populations (PAHO, 2017). The diagnosis of CKDu is difficult in field studies due to the need for consistent monitoring of physiological changes over several months. However, a risk factor that has been recently recognized as an important cause of the development of CKD is acute kidney injury (AKI), especially recurrent incidents of AKI (Chawla and Kimmel, 2012). AKI events can be detected on the basis of elevations in serum creatinine that occur rapidly and are detectable within hours or days (Ronco, 2013). Similar to CKDu, occupational and environmental risk factors in agricultural work settings, such as pesticide exposure and repeated episodes of heat stress accompanied by dehydration, may increase the risk for AKI in farm workers (Correa-Rotter et al., 2014; Levin et al., 2017; Wesseling et al., 2014). Thus, a few studies have begun evaluating the incidence of AKI in agricultural settings and found that heat stress accompanied by dehydration may be associated with AKI (Butler-Dawson et al., 2019; García-Trabanino et al., 2015; Moyce et al., 2016). Analyzing kidney functioning at earlier stages is crucial to prevent kidney damage, so recent studies have investigated the early decline of the estimated glomerular filtration rate (eGFR) in relation to risk factors that affect kidney function before a possible injury (Butler-Dawson et al., 2018; Sorensen et al., 2019).

While most studies related to CKD and AKI have focused on workload, heat stress and hydration, the combination of other potential risk factors such as dietary fructose consumption, heavy metals and agrochemicals remains fully unevaluated (Coca et al., 2012; Lucas et al., 2020). There is a need for studies that can evaluate occupational risk factors in combination with heat stress and dehydration. For example, pesticide exposure has been linked to kidney dysfunction in animal studies, but there is a lack of epidemiological studies to support this (Scammell et al., 2019). Further, most epidemiological studies are based on self-reported pesticide exposures and do not include direct pesticide measurements in either the environment or biological samples (Scammell et al., 2019; Wegman et al., 2016; Wesseling et al., 2014). More studies are needed to properly assess possible interactions between heat stress and pesticide exposure as it relates to kidney function in the workplace (Valcke et al., 2017).

In 2016, we conducted a pilot study to evaluate exposure to heat and pesticides in 20 migrant seasonal farm workers (MSFWs) working at a large-scale grape farm during the harvest season in the semi-arid region of Sonora, Mexico. This region which borders the U.S. state of Arizona, is considered approximately 95% arid or semi-arid land and is characterized by a lack of precipitation and extremely high temperatures (Hallack-Alegria and Watkins Jr, 2007). Working in large-scale commercial farms in Sonora, MSFWs conduct strenuous tasks while exposed to a wide array of occupational risks and hazards, such as extreme heat and pesticide exposure. We found that nearly all MSFWs reported working in extreme heat, most had core body temperatures reaching and exceeding 38 °C at some point during the workday, and all workers were dehydrated to some degree (Wagoner et al., 2020). Also, many had high levels of organophosphate (OPs), pyrethroids (PYRs), and imidacloprid biomarkers in their urine; the highest pesticide levels were found during the summer, late in the harvest season (Lopez-Galvez et al., 2018; López-Gálvez et al., 2020). Although the MSFWs are exposed to occupational risk factors such as heat and pesticides, to our knowledge, no studies have comprehensively examined, longitudinally, the potential effect of these combined occupational factors on kidney function in MSFWs. Therefore, the objective of this study was to longitudinally assess the kidney function in MSFWs as a function of pesticide exposure, heat stress and dehydration in the Northern agricultural region of Sonora, Mexico over an entire grape harvest season.

## 2. Methods

In this study, we evaluated the longitudinal changes in kidney function in MSFWs (n = 101) and office workers (i.e., the reference group, n = 50). We also evaluated the association between heat strain, dehydration, pesticide exposure and kidney function.

### 2.1. Location and study population

This longitudinal study was conducted over a six-month period in the year 2019 on a large commercial table-grape farm in Sonora, Mexico. The agricultural company contracts approximately 2000 MSFWs per year to work on the farm during for the pre-harvest and harvest season. This large table-grape farm conducts both organic and conventional farming methods in separated designated fields. The conventional field areas of the farm are commonly treated with pesticides and artificial fertilizers, whereas the organic farming takes place in separate United States Department of Agriculture (USDA)-certified organic field areas with approved pesticides and fertilizers. The on-site agricultural engineers informed our research team that apart from the application of certain agrochemicals, the farming techniques between the organic and conventional areas are very similar to each other. The artificial pesticides applied only in the conventional area during pre-harvest included a variety of fungicides (Triazoles) and insecticides (OPs, PYRs, and Imidacloprid), while a few herbicides and insecticides (Imidacloprid and PYRs) were applied during the harvest season. Most of the agrochemicals applied in this farm are injected through the drip irrigation system.

The study population included MSFWs who were bused from their home states to work as field workers in this farm. MSFWs (n = 101) were enrolled in the study upon their arrival while participating in the general registration conducted by the agricultural company who hired them. Only the MSFWs, who worked in the fields and who do not directly apply or mix pesticides were recruited for this study. To evaluate exposure to pesticides, 50 MSFWs were randomly selected to work in the organic certified area of the farm and 51 MSFWs were randomly selected to work in the conventional area. Randomization occurred via the placement of several cards with two colors (green and black) in a box and asking participant to grab one card from the box without looking. Participants who picked the green card were assigned to the organic field areas and those who picked the black card were assigned to the conventional field areas. It is important to mention that all MSFWs lived on the farm in shared dormitories provided by the company, shared the same physical space while eating meals, and were served the same food via an on-site cafeteria provided by the company, as workers did not have kitchens in their dormitories. There was no division of dormitories for participants working in the conventional or organic area. The agricultural company also provided well water from the farm to all MSFWs. Apart from lunch breaks, there are no other mandatory breaks; however, the company provides resting areas without shade, portable toilets with hand washing stations, and large tanks with hauled water placed on the field at end of the grapevine rows. The MSFWs conducted similar field work activities regardless of the type of field (conventional or organic) for about 10–12 h a day. During the pre-harvest, MSFWs worked on training grapevines in addition to thinning of grape clusters. They also pruned plants and cleaned grapevines. During harvest, MSFWs carried plastic bins to put the hand-harvested grapes and then weighed them at stations located at the end of grapevine rows.

In addition, office workers (n = 50), who were permanent residents of Sonora and worked for the same agricultural company in a town located 30 min away from the farm, were recruited as a non-farmworker reference group. All of the study participants in this study were male because about 90% of the workers hired by this agricultural company are males. Sampling and survey administration of participants was conducted twice: at the pre-harvest (i.e., the beginning of March 2019) and in the late harvest (i.e., July 2019). Participants with previous diagnoses of renal health issues, diabetes or hypertension were excluded from the study. The medical examination/screening was conducted by an on-site medical team that included three medical doctors provided by the agricultural company. Written consents in Spanish were obtained from all participants. The University of Arizona Human Subjects Protection Program reviewed and approved all study procedures (IRB approval protocol number: 1810987689R001).

### 2.2. Questionnaires

All participants were surveyed to collect information on demographic and behavioral characteristics such as age, primary language, state of origin, income, educational level, smoking and alcohol consumption, and current medication usage. Additional questions related to typical hydration practices (e.g., amount of water and sweetened beverages consumed per day), and pesticide exposure (e.g., application of insecticides inside their home or dormitories were asked of all participants after their workday at each sampling timepoint (i.e., early, and late harvest). With respect to water consumption, since all MSFWs carried water in a 1 L plastic jug during their workday in the field, we asked each worker how many of those water jugs did they drink that day. All surveys were administered orally in Spanish by trained member of our research team.

### 2.3. Heat stress, physiological strain index, and anthropometric measurements

Heat stress was assessed using the wet bulb globe temperature (WBGT) index by continuously collecting the following environmental measurements¶: globe temperature (*T*_*g*_), dry air temperature (*T*_*a*_), relative humidity (*RH*), and wet bulb temperature during the entire study period using a REED Heat Index WBGT Meter (model SD-2010; REED Instruments, Wilmington, NC). One monitor was placed near the grape fields to estimate MSFWs heat stress and a second monitor was placed inside the office space of the reference group to estimate office workers’ heat stress levels. The WBGT was monitored for the organic and conventional grape fields near workers’ daily activities. Each monitor was attached to a tripod at a height of 1.6 m at each site. Monitors were set to continuously collect data every minute for the length of the study. The effective WBGT (WBGT_eff_) was calculated by incorporating the ACGIH clothing correction factor to the 8-h average WBGT for each participant (Bernard et al., 2007).

Participants’ heat strain was determined using the physiological strain index (PSI) developed by Moran et al. (1999), which was calculated using core body temperature at rest (Temp resting) and at work (Temp working) and heart rate at rest (HR resting) and at work (HR working) as presented in equation (1) (Moran et al., 1999). Core body temperature was estimated from the ear canal temperature measurements, which were collected using a digital thermometer (Braun Thermoscan ExacTemp model IRT 6520; Braun, South Boston, MA). Heart rate was collected using a digital sphygmomanometer (Omron M10-IT, Omron Healthcare Inc., Bannockburn, USA). Each of these measurements were taken from each participant twice: first, a half an hour before their work shift began and second, about 3 h after their work shift began. It is important to mention that although HR and ear temperature measured at work could be affected by workers temporally stopping their activities during sampling, our trained research team collected these measurements on the field in a short time, less than a minute per person, to capture the heat strain of their work-shift.

(1) $$PSI=\frac{5(T\text{ working }-T\text{ resting })}{39.5-T\text{ resting }}+\frac{5(HR\text{ working }-HR\text{ resting })}{180-\;HR\text{ resting }}$$

Other anthropometric measurements such as height, weight, and blood pressure were collected from each participant in the morning before their work shift at both pre-harvest and late-harvest sampling points. A stadiometer (Seca, Birmingham, UK) was used to measured participants’ height, while their weight was measured with minimal clothing using a Seca 813 digital flat scale (Seca, Birmingham, UK). Systolic and diastolic pressure were measured using a digital sphygmomanometer (Omron M10-IT, Omron Healthcare Inc., Bannockburn, USA). Age, heart rate and body weight were used to calculate metabolic work rate (W/m^2^) by using the standard ISO 8996 equation (Bröde and Kampmann, 2018; ISO, 2017). Body mass index (BMI) (kg/m^2^) was calculated by dividing their body weight by the squared height of each participant. Total body water was estimated from weight and height measurements using the Boer, 1984 formula, as presented in equation (2) (Boer, 1984).

(2) TBW=0.297(Weight)+19.5(Height)−14.0

### 2.4. Urine and blood sampling

Participants were instructed to collect their first morning void urine sample in a 120 mL sterile polyethylene plastic container in the morning after the observed work shift. Collection of first morning void is highly recommended as the body fluid compartments are equilibrated providing a more uniform assessment (Cheuvront et al., 2015; Hamouti et al., 2010; Johnson et al., 2013). Additionally, blood samples were drawn through venipuncture from all participants by an on-site medical doctor using 4 mL BD Vacutainer blood collection tubes (Becton Dickinson & Co., USA). The biological samples were transported on ice the same day in a cooler to a locally certified clinical laboratory (Labo-ratorio Bio-Clinico Martinez, Hermosillo-Sonora, Mexico) to be processed the same day as sample collection. The biological samples from all participants were collected the morning after a normal workday for each study timepoint (i.e., pre-harvest and late harvest). It is important to mention that for the MSFWs group, the collection of biological samples, heat strain, metabolic work rate, and other anthropometric measurements corresponded to the first day of work during the pre-harvest, and a regular workday during the late harvest.

### 2.5. Laboratory analysis

Urinary specific gravity was determined with dipstick analyses of the urine samples, using Bayer Clinitek Chemistry Analyzer with Multistix 10SG reagent strips (Siemens Diagnostics, United States). The blood samples were centrifuged at 3500 RPM for approximately 10 min to separate the serum from red blood cells. Samples were analyzed within a few hours of collection. The creatinine (Cr) and uric acid in serum samples were analyzed using a single-slide enzymatic method, the VITROS CREA and the URIC slide method with the VITROS Calibrator Kit 1, which uses three levels of calibration to obtain quantitative measurements read by the automated VITROS 350 Chemistry Systems analyzer (Ortho Clinical Diagnostics, Rochester, NY). The serum osmolality (mOsm/L) was calculated from the sodium, blood urea nitrogen (BUN), and glucose in serum, which were measured using an enzymatic method with a fully automated VITROS 350 chemistry system analyzer (Vitros Na, BUN, GLUC; Ortho Clinical Diagnostics, Rochester, NY). After the laboratory analyses, remaining urine was transferred to 15 mL polyethylene tubes and remaining blood serum was transferred to cryogenic vials and transported on ice to be stored at −80 °C at the University of Arizona.

### 2.6. Data analysis

The data were entered into Microsoft Excel (2007) and organized in IBM SPSS (Version 24). The data were then analyzed using Rstudio software version 3.5.1 (Team, 2015). We estimated the glomerular filtration rate (eGFR) from serum creatinine (SCr) using the Chronic Kidney Disease Epidemiology Collaboration (CKD-EPI) equation developed by Stevens et al. (2011), in which a four level race variable allowed us to include Latino and Indigenous groups in the calculation presented in equations (3) and (4)(Stevens et al., 2011).

(3) $$For\;SCr\leq 0.90\;mg/dL:\text{eGFR}=143\times 0.993^{Age}\times {\left(SCr/0.9\right)}^{-0.412}$$

(4) $$For\;SCr>0.90\;mg/dL:\text{eGFR}=143\times 0.993^{Age}\times {\left(SCr/0.9\right)}^{-1.210}$$

Descriptive statistics were calculated for demographic and occupational characteristics. Differences between MSFWs and office workers at pre-harvest were assessed using a *t*-test for continuous variables and chi-square test for categorical variables. Variable distribution was examined using histogram and summary statistics. Variables with a lognormal distribution were log-transformed prior to analyses. Both MSFWs and office workers were included in the analyses to examine changes over time. The differences among variables between the two sampling time points, pre-harvest (March) and late harvest (July), were assessed with paired t-tests. In order to evaluate several factors of interest associated with eGFR, we performed linear mixed effects regression analyses with random intercepts for individuals to account for the repeated measures on the same participant using the data collected during pre-harvest and the late harvest timepoints. Occupational factors, individual behaviors and demographic characteristics known to be risk factors in reduced kidney function were selected *a priori* based on recent literature and data collection feasibility (Butler-Dawson et al., 2019; Moyce et al. 2018, 2020; Sorensen et al., 2019).

We used simple linear mixed effect regression models to independently evaluate each individual variable effect on eGFR. The independent variables that were statistically significant in the simple linear mixed regression analyses were considered for adjusted linear mixed effect models. To select variables for inclusion the models, the significance levels of the simple regression analyses were set with a p-value < 0.2. Four linear mixed effect models were built based on the main exposures of interests (heat strain, dehydration, and job category as a proxy to pesticide exposure). To evaluate differences between eGFR at pre-harvest and late harvest, we added time as the main variable of interest in our first adjusted linear mixed model. Our second model included only heat strain, water intake and dehydration as main variables of interest. In model 3, we assessed the association of pesticides and eGFR by including job category and pesticides used at home as a proxy for pesticide exposure. The differences by job during harvest season were evaluated by including an interaction term between time and job category in model 3. In the final adjusted model (4th model), all variables of interest, including PSI, dehydration and job category were included. Models were fit with interaction terms of each variable of interest. Variables such as age, BMI, and blood pressure were included in all adjusted models (Laws, 2015). Observations with missing values (19.9% of the dataset had missing values in this study) were imputed using Multivariate Imputation by Chained Equations (MICE) (Buuren and Groothuis-Oudshoorn, 2010). We also conducted a sensitivity analysis, where a reduced eGFR model was computed one more time by using only the complete data (without missing values as all participants datapoints with incomplete observations were deleted). Each set of variables were evaluated for multicollinearity. The correlation coefficients and variance inflation factor (VIF) were used to assess multicollinearity. Independent variables with a significant correlation coefficient >0.7 or a VIF >2 were removed one by one from models (Dohoo et al., 1997; Dormann et al., 2013).

## 3. Results

### 3.1. Study population

Before consenting participants, we screened out potential participants (17 MSFWs and 2 office workers) who had been previously diagnosed with hypertension or diabetes. In terms of study attrition, out of the 151 recruited participants, 26 MSFWs (17% of the total population, 13 from each field group, organic and conventional) dropped out of the workforce before the end of the harvest and did not participate in the final study measurement. An additional 30 office workers (19% of the total population) were absent during the final study measurement. Although this study has a total attrition rate of 36%, there were no significant differences between the demographic characteristics and measurements of the MSFWs who completed the study versus the ones who dropped out before the late harvest (Table S1–S2 in Supplementary materials). Regarding the attrition of the office workers, unfortunately, the participants that were more likely to drop out were older participants, with a higher BMI and higher systolic blood pressure than their counterparts who completed the study. In terms of kidney function, while the average eGFR and serum creatinine measured in office workers who completed the study were different than in office workers who did not complete the study, these differences were not statistically significant (Table S3, Supplementary materials).

### 3.2. Demographic, occupational and behavioral characteristics

As presented in Table 1, participants’ ages ranged from 18 to 59 years, with an average of 28.5 years. Although there were no significant differences between the ages of MSFWs and office workers, MSFWs were slightly older than office workers. There were significant differences in workers’ primary language, as 34% of MSFWs reported an Indigenous language as their primary language, compared to only 10% of the office workers. In terms of workers’ state of origin, most MSFWs came from Puebla (55%) and Chiapas (41%), while most office workers were from Sonora (80%). There were significant differences in the educational level and monthly income acquired by workers, as only 15% of the MSFWs had received at least some high school education and most (86%) earned less than $500 per month. On the other hand, 40%the office workers earned more than $500 per month. And most office workers (86%) received a high school education and above. Smoking and recent consumption of alcohol was significantly higher in MSFWs than in office workers, as almost half (46%) of MSFWs reported smoking and most (66%) reported having recently consumed alcohol. This is an important contrast to the office workers, as only 22% reported smoking and less than 1/3rd (32%) reported recent consumption of alcohol. There were no significant differences in the use of pain medications reported at baseline between MSFWs and office workers.

In terms of hydration practices, most participants (86.8%) reported consuming at least 4 L of water per day at baseline and there were no significant differences in reported daily water intake between MSFWs and office workers. Most participants (66.5%) reported having recently (same day that the questionnaires were administered or one day ago) consumed soda and 40% reported consuming at least one soda per day. There were significant differences in both the frequency and amount of soda consumed between MSFWs and office workers (p < 0.001). Recent consumption of soda was significantly higher in MSFWs compared to office workers, as 77.3% of MSFWs recently consumed soda compared to only 44% of office workers. Also, 24.8% of MSFWs consumed more than one soda per day compared to office workers (2.0%). Finally, although most participants reported not recently applying insecticide in their homes or dormitories, significantly more MSFWs (24.8%) applied insecticides in their dormitories, compared to 10% of office workers who applied insecticides in their homes.

### 3.3. Pre-harvest measurements of the studied population

As shown in Table 2, the average participants’ body weight was 71.0 ± 12.5 kg and there was no significant difference in body weight between MSFWs and office workers. There was a significant difference in participants’ height, as MSFWs were shorter on average 1.64 ± 0.6 m, compared to office workers, 1.73 ± 0.7 m (p < 0.001). MSFWs also had a significantly higher average BMI, 26.6 ± 3.9 kg/m^2^, compared to office workers, 23.7 ± 4.3 kg/m^2^ (p < 0.001). Half of the MSFWs were considered overweight and 15% were considered obese, while most officer workers (66%) had a normal BMI. The average systolic and diastolic blood pressure among all participants was 115.1 ± 17.5 mmHg and 73.2 ± 13.1 mmHg, respectively. Blood pressure was not significantly different between MSFWs and office workers at baseline.

In terms of work intensity, the mean metabolic work rate was significantly higher in MSFWs, 169 ± 43.1 Watts/m^2^, compared to office workers, 133 ± 29.8 Watts/m^2^ (p < 0.001). In addition, heat stress exposure was significantly higher in MSFWs than in office workers, as the average-8hr WBGT_eff_ for MSFWs was 17.7 ± 3.4 °C compared to 13.7 ± 1.11 °C for office workers. There was also a significant difference between MSFWs’ and office workers’ PSI levels, as the median PSI for MSFWs and office workers were 2.7 and 1.8, respectively (p < 0.001). The mean total body water was significantly higher in officer workers (41.0 ± 5.2 mL/kg) compared to MSFWs (38.8 ± 3.9 mL/kg). With respect to dehydration, the average serum osmolality across all participants was 264.0 ± 17.4 mOsm/kg and there was not a significant difference between the two participant groups at the pre-harvest sampling period.

Similarly, even though about half of the participants (49.7%) were dehydrated, with urinary SG greater than 1.021, there were no significant differences in the SG between MSFWs and office workers. For the kidney functioning, the median serum creatinine was 0.70 mg/dL and the average eGFR levels were 126 ± 14.2 mL/min/1.73 m^2^ across all participants. There were no significant differences in serum creatinine or eGFR between MSFWs and office workers at pre-harvest. Also, there were no significant differences in the characteristics and measurements collected at pre-harvest between the MSFWs who worked in the organic field area versus the MSFWs who worked in the conventional field area (Table S4–S5, Supplement materials).

### 3.4. Pre-harvest to late harvest changes in kidney function and other physiological measurements

**Fig. 1.**
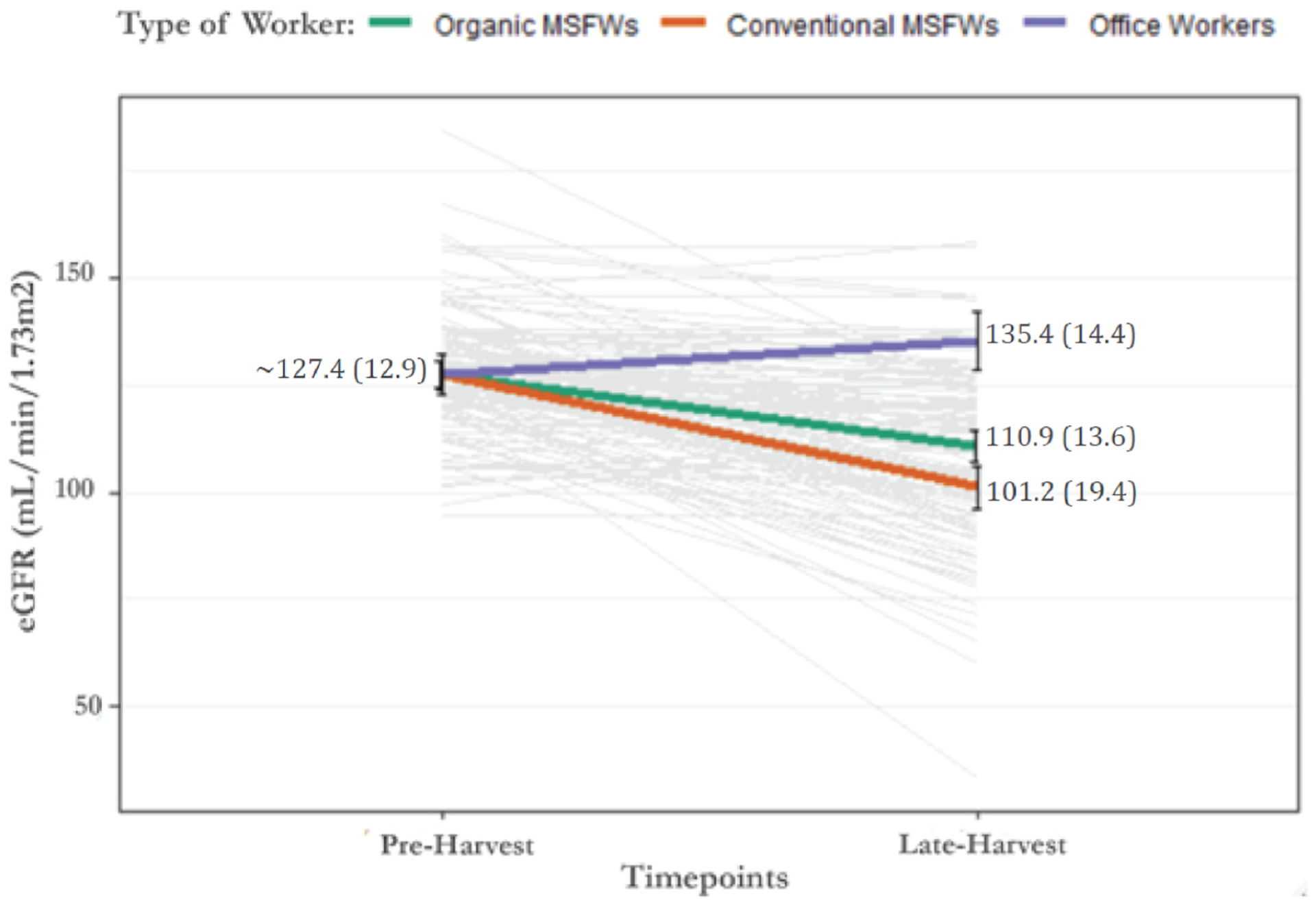
Participants’ eGFR (mL/min/1.73 m^2^) by job categories, office workers versus MSFWs in organic fields versus MSFWs in conventional fields, during pre-harvest and late harvest timepoints.

Although there were no participants with kidney function issues at pre-harvest, by the late harvest, one MSFW (in conventional field) experienced an 87% decline in eGFR with levels below 60 mL/min/1.73 m^2^, which can be consider as kidney disease. Also, based on the Risk/Injury/Failure/Loss/End-stage (RIFLE) criteria for acute kidney injury classification, two of the MSFWs who worked in the conventional field developed a kidney injury documented by a decreased of eGFR greater than 50% by the late harvest (Table 3) (Bellomo et al., 2004). In addition, by the late harvest, 12 participants, all of whom were MSFWs (8 working in the conventional field and 4 in the organic field), experienced a decrease in eGFR greater than 25%, putting them at risk of kidney injury (Table 3). While no participants had eGFR levels below 90 mL/min/1.73 m^2^ at the pre-harvest, eight MSFWs had a significant decline in eGFR levels (under 90 mL/min/1.73 m^2^, an indication of kidney damage) from pre-harvest to the late harvest; all eight (21%) worked in the conventional field (Table 4). Also, by the late harvest, one MSFW, who worked in the conventional field, experienced a decline in eGFR of 87% with levels below 60 mL/min/1.73 m^2^, an indication of kidney disease (Table 4). The mean eGFR among MSFWs decreased significantly from 125 ± 13.0 mL/min/1.73 m^2^ at pre-harvest to 106 ± 17.5 mL/min/1.73 m^2^ at late harvest (Fig. 1). Comparatively, the mean The Risk, Injury, Failure, Loss of Kidney Function and End Stage Kidney disease (RIFLE) classification. eGFR in office workers increased from 127 ± 16.6 mL/min/1.73 m^2^ to 135.4 ± 14.4 mL/min/1.73 m^2^, though this increase was not significant (Fig. 1). The results of the measurements taken from MSFWs who worked in the organic field or conventional field areas throughout the study are shown in Table 5. A significant decline in eGFR levels was observed for MSFWs who worked in the organic field area from pre-harvest to late harvest (127.5 ± 12.2 and 110.9 ± 13.6 mL/min/1.73 m^2^, respectively) (Table 5, Fig. 1). Similarly, as shown in Table 5 and Fig. 1, the eGFR levels decreased significantly in MSFWs who worked in the conventional area from pre-harvest to late harvest (127.4 ± 12.9 and 101.2 ± 19.4 mL/min/1.73 m^2^) (p < 0.001). No eGFR differences were observed between the two MSFW groups at the pre-harvest; however, during the late harvest, the mean eGFR was significantly higher in MSFWs who worked in the organic field (110.9 ± 13.6 mL/min/1.73 m^2^), compared to MSFWs who worked the conventional field (101.2 ± 19.4 mL/min/1.73 m^2^) (p = 0.002). No significant changes in eGFR were observed in office workers from pre-harvest to late harvest (Table 5).

Regardless of whether MSFWs worked in the organic or conventional field, the serum creatinine and other blood biomarkers, including uric acid, BUN, and osmolality, increased significantly from pre-harvest to late harvest (Table 5). Blood biomarkers were not significantly different in office workers from pre-harvest to late harvest. Systolic blood pressure increased significantly from pre-harvest to late harvest in MSFWs working in the organic, as well as MSFWs working in the conventional field, but there was not a significant change in diastolic blood pressure during these two timepoints. There were no significant differences in the systolic blood pressure between MSFWs working in the conventional versus organic area during any of the sampling timepoints. Also, there were no significant changes in the diastolic or systolic blood pressure observed in office workers from pre-harvest to late harvest. There were, however, significant decreases in BMI from pre-harvest to late harvest in office workers and in MSFWs working in the conventional field. Similarly, the total body water (TBW) decreased significantly in MSFWs working in the conventional field and in office workers. The significant decline in BMI and TBW measured in office workers cannot be explained. For MSFWs working in the organic field, their BMI and TBW decreased, but not significantly, during the study (Table 5). While there were no significant changes in office workers’ metabolic work rate and PSI during the two study time points, the metabolic work rate and PSI increased significantly in MSFWs working in both the organic field and conventional fields from pre-harvest to late harvest (Table 5, Figure S1 in Supplementary materials).

### 3.5. Regression models

As presented in Table 6, in our first model the variable time was the main independent variable of interest, adjusted for covariates which were selected based on their significance (p < 0.2) in simple linear mixed effect analyses. After adjusting for education, BMI, age, and systolic blood pressure, a significant decrease of 16.98 mL/min/1.73 m^2^ (95% CI: −20.22, −13.69) in eGFR was observed among participants during the late harvest timepoint compared to the pre-harvest timepoint.

Our second model incorporated the primary variables of PSI, self-reported water intake, and dehydration based on SG (Table 6, *Model 2*). In a post-hoc analysis, the variables for time, metabolic work rate and WBGT_eff_ were excluded from the second model (Table S8a–d; Figure S3.1 in Supplementary materials). After controlling for education, age, BMI, and systolic blood pressure, we found that for every 1% increment in PSI, there was a significant decrease in eGFR of 0.16 mL/min/1.73 m^2^ (95% CI: −0.15, −0.06). In addition, the association between dehydration and eGFR remained strong, as participants with mild dehydration during the study period had a significant decrease in eGFR of 9.68 mL/min/1.73 m^2^ (95% CI: −13.31, −3.26) compared to hydrated participants. A significant decrease of 13.62 mL/min/1.73 m^2^ (95% CI: −16.56, −7.71) in eGFR was observed in dehydrated participants compared to hydrated participants. Although there was a decrease of eGFR among participants who consume more than 4 L of water, the significance disappeared after adjusting for covariates (Table 6, *Model 2*).

In the third model, as presented in Table 6, the primary independent variables of job category and insecticide application inside homes/dormitories were adjusted by the following covariates: age, BMI, and systolic blood pressure. After examining for multicollinearity, education was excluded from the third model (Table S8a–c; Figure S2 in Supplementary materials). There was a significant difference in the effect of time by job category, so an interaction term between time of sampling (pre-harvest and late harvest) and job category was included in this model (Table S9, Supplementary materials). Thus, from early harvest to late harvest, there was a significant decrease in eGFR of 19.02 mL/min/1.73 m^2^ (95% CI: −27.29, −10.82) among MSFWs who worked in an organic field compared to office workers. Also, in comparison to office workers, a significant decrease of 29.17 mL/min/1.73 m^2^ (95% CI: −37.26, −21.17) was observed in MSFWs who worked in the conventional field throughout the study time points. Even though a decrease in eGFR was observed among participants who reported recently applying insecticide in their home/dorms, the significance of the associations between eGFR and insecticide application disappeared after covariates were included (Table 6, *Model 3*).

In the final model (*Model 4*) presented in Table 7, the main variables of interest were PSI, job category, dehydration, self-reported water intake and recent home/dorm insecticide application. Due to multicollinearity, time and education were excluded from the final model. We found a significant interaction term between PSI and job category, so we included this interaction term in the final model (Table 7). After controlling for BMI, age, systolic blood pressure, hydration and insecticide application at home, a 1% increment in PSI among MSFWs who worked in the organic field, was associated with a significant decrease of 0.23 mL/min/1.73 m^2^ (CI 95%: −0.41, −0.03) in eGFR when compared to office workers. Also, in comparison to office workers, a 1% increment in PSI among MSFWs who worked in the conventional field was associated with a significant decrease of 0.37 mL/min/1.73 m^2^ (95% CI: −0.57, −0.16) in eGFR. With respect to participants’ hydration levels in the final model, there was a significant decrease in eGFR of 8.51 mL/min/1.73 m^2^ (95% CI: −13.98, −3.03) among participants who had mild dehydration and an eGFR decline of 11.99 mL/min/1.73 m^2^ (95% CI: −16.88, −7.10) among dehydrated participants in comparison to participants who were normally hydrated at each timepoint. The associations between job category and eGFR disappeared after controlling for all covariates including heat and hydration. There were no significant associations between self-reported water intake and insecticides application at home/dorm in the final model with eGFR (Table 7). This final model was also conducted with serum creatinine as the outcome variable, and the results from that analysis indicated that the same variables associated with the decline of eGFR are also significantly associated with the increase of creatinine during the study (Table S11, Supplementary materials).

### 3.6. Sensitivity analysis

We found that the results of this sensitivity analysis are similar to the MICE imputed model. As presented in Table S12 (Supplementary materials), most of the variables, except for mild dehydration, that were found to be significant in the MICE model, remained significant in the reduced model (deleted missing values). The final R^2^ between these two models were also very similar to each other.

## 4. Discussion

To our knowledge, this is the first longitudinal study to assess kidney functioning in relation to heat strain and pesticide exposure by evaluating MSFWs who work in organic and conventional fields and comparing them with office workers as a reference group. The main findings of this study are that kidney function among MSFWs decreased significantly from pre-harvest to late harvest, while no significant change in kidney function was observed in office workers. Although all workers had a normal kidney function at baseline, as MSFWs worked in the grape fields over the course of several months, they experienced a significant decline in kidney function by late-harvest. In fact, one MSFW developed a kidney disease and two MSFWs experienced a kidney injury by the end of the study, however, further clinical measurements are necessary to confirm any kidney disease development. It is important to mention that no unique behavioral or personal factors were observed in these MSFWs during the study that may have resulted in extreme decline of the eGFR levels. Also, we found that by the late grape harvest, a total of 14 (19%) MSFWs were at high risk of suffering a kidney injury, as their eGFR declined by more than 25% from pre-harvest to late harvest. On the other hand, no office workers were at high risk of developing kidney injury during the study. We found that all MSFWs that experienced a decline in eGFR below 90 mL/min/1.73 m^2^ by the late-harvest worked in the conventional field, while no office worker or MSFWs in the organic field had eGFR levels below 90 mL/min/1.73 m^2^ during the study. These results may indicate that the prolonged exposures to occupational hazards in grape field workers, especially for the ones working on conventional fields in this region, can experience a significant impair of their renal function in comparison to non-field workers.

There were no significant differences between blood pressure, dehydration levels, and kidney function biomarkers including eGFR between MSFWs and office workers at pre-harvest. However, during the study period, only MSFWs experienced a significant increase of systolic blood pressure, serum creatinine, BUN, serum uric acid, and serum osmolality. This suggests that as MSFWs work on the grape farm into the hotter months of the harvest season, their health was negatively impacted, compared to office workers. For example, the significant increase of uric acid in serum, which can be increased after strenuous physical activity and potential muscle injury, observed in MSFWs can lead to harmful conditions such as the development of hyperuricemia (Ames et al., 1981; Arakawa et al., 2016; Suzuki et al., 2006). Hyperuricemia has been previously associated with the increase of eGFR in sugarcane workers in Guatemala, as excess serum uric acid can alter the regulation of the renal perfusion pressure, which can increment the glomerular pressure (Sorensen et al., 2019). In our study, we also found that the increment of uric acid in participants during the harvest season is significantly associated with a decrease of eGFR. Thus, monitoring of uric acid in MSFWs may be used as an early indicator of kidney injury.

### 4.1. Heat strain and dehydration

There was a significant association between heat strain, measured by PSI, along with the participants’ dehydration and kidney functioning decline during the study timeline. The high temperatures coupled with the intense physical workload can have a physiological effect on the body related to kidney function. Our results of the simple linear mixed models indicate that heat stress, heat strain measured by PSI, and metabolic work rate measurements along with water intake and dehydration levels were significantly associated with a decline in eGFR over the study timeline. The association between PSI and decline in eGFR continued to be significant after controlling for water intake, dehydration, education, BMI, age, and systolic blood pressure, as presented in our *model 2*. This indicates that heat strain may affect workers’ kidney function. Although a few studies have suggested that heat stress in combination to physical workload are associated with AKI and the development of CKD (Butler-Dawson et al., 2019; Paula Santos et al., 2015; Sorensen et al., 2019; Wesseling et al., 2016), only one other study conducted in California, U.S. has utilized PSI to evaluate workers’ heat strain levels in relation to changes in eGFR (Moyce et al., 2018). Similarly, Moyce et al. (2018) also found that heat strain (i.e., PSI) in agricultural workers was associated with decreased kidney functioning.

In addition, we found that dehydration played an important role in the decrease of eGFR. As presented in the *model* 2, dehydrated participants, measured with SG, had significantly lower eGFR levels compared to better hydrated participants. Our results support the idea that dehydration is a risk factor for kidney damage among workers exposed to hot environments while working (Roncal-Jimenez et al., 2015). Furthermore, in terms of water consumption, there was a significant association between an increase in water consumption and a decline in eGFR. This association may indicate that participants drink more water with the increment of workload, not necessarily as a preventative measure. Nonetheless, this association was not evident after controlling for PSI and other covariates in *model 2*. Our results on water consumption and eGFR are consistent with other studies in which an increase on self-reported water consumption is associated with eGFR decline (Laws, 2015; Sanoff et al., 2010; Wesseling et al., 2016). This supports the idea that perhaps the workers who drank large amounts may be compensating for working hard (Moyce et al., 2020). It is important to consider the effect of hyperthermia alone or in combination with dehydration, as consequences of the strenuous physical work conducted during high levels of heat stress, which can lead to a reduced renal blood flow (Cotter et al., 2014; Cuddy and Ruby, 2011). The renal blood flow decreases because the blood is redirected to the working muscles during the physical activity and to the skin, as a heat dissipator; thus, the ability of the kidneys to eliminate waste products is reduced (Hansson et al., 2020). We also found that recent (within the last 2 days) consumption of soda among participants was significantly associated with decreased eGFR. This suggests that the intake of sugary drinks could be an important kidney disease risk factor, as previous experimental studies have shown that rehydration with sugary drinks containing fructose can accelerate kidney issues (Schlader et al., 2019; Sánchez-Lozada et al., 2019). However, the association between recent consumption of soda among participants and lower eGFR disappeared after controlling for PSI. The lack of an association with soda consumption and eGFR is similar to the results reported by a recent study conducted in California among agricultural workers, in which there was no association between sugary drinks and AKI (Moyce et al., 2020).

### 4.2. Job categories as proxy to pesticide exposure

As shown in *model 3*, we found a significant decrease in kidney functioning over time (from pre-harvest to late harvest) among MSFWs who worked in the organic field and MSFWs who worked in the conventional field compared to office workers. This decrease in eGFR was most prominent in MSFWs who worked in the conventional field. The association between both MSFW job categories (organic and conventional) and the decrease of eGFR was strengthened after controlling for the application of pesticides in home, BMI, age, and systolic blood pressure. Our findings suggest that field workers who are potentially exposed to pesticide at work could be at a high risk of having lower kidney function and developing kidney injury. Even though no previous study has compared kidney function among workers in organic versus conventional fields, other studies have found a significant increase in serum creatinine and/or a decrease in eGFR in farm workers who self-reported to have applied or mixed pesticides (García-Trabanino et al., 2015; Hassanin et al., 2018; Jayasumana et al., 2015; Lebov et al., 2016).

It is important to note that none of the participants, MSFWs or control groups alike, applied pesticides at work directly. For example, though MSFWs working in the conventional field may have been exposed to pesticides, they themselves were not responsible for applying the pesticides. Thus, we could expect worse outcomes for farmworkers that are involve in direct pesticide application; also, further studies should be conducted on field workers (non-pesticides applicators), as indirect exposure to pesticides in the field may affect their kidney function Additionally, even though we did not observe an association between using insecticides inside the home/dorm and eGFR in our final model, more research is needed, as farm workers could have double risk of exposure at work and home in comparison to other occupations where they are only exposing themselves by choice in their home.

### 4.3. Heat strain-hydration and pesticides

In our final model, which was adjusted for heat strain, dehydration, and pesticide proxy variables, we found that the workers’ heat strain, estimated by PSI, and dehydration were significantly associated with the decrease of eGFR, while job categories (used as a proxy for pesticide exposure) and recent use of insecticides within their home/dorm were no longer significantly associated with eGFR. These results are consistent with many studies which support the importance of heat stress and hydration as main factors that affect kidney function in farm workers (Butler-Dawson et al., 2019; Laws, 2015; Moyce et al. 2016, 2018). Thus, as shown in other studies, reduction of heat stress in addition to rehydration with electrolytes may protect workers in this region from future kidney injuries (Wesseling et al., 2016).

Additionally, we found an important interaction between heat strain and job category, a proxy variable to pesticide exposure, as the decrease in kidney functioning by job category or pesticide exposure was related to the increment in heat strain. The interaction we documented, supports the idea that pesticide exposure should be analyzed in combination with heat stress and dehydration in relation to kidney function (Valcke et al., 2017). Some experimental studies have demonstrated that the combination of these factors can have synergistic negative health effects in mice (Gordon et al., 2014; Leon, 2008), but the potential synergistic effects caused by heat stress and pesticides has not been fully evaluated in epidemiological studies. Also, the absorption and toxicity of pesticides in the body may be exacerbated with the increment of heat stress and core body temperature (Gordon et al., 2014). As discovered in our previous pilot study on this farm, the high levels of pesticide metabolites in urine were correlated with heat stress and body temperature (López-Gálvez et al., 2020), which can potentially affect kidney function. Further research should be conducted in the interaction of heat and pesticides in relation to kidney functioning.

The changes in kidney functioning that we found by job category due to heat strain are not only consistent with the hypothesis that heat stress may be a main contributing factor of developing a kidney disease, but it suggests that pesticide exposure is a potential factor that needs to be consider in conjunction with heat stress and dehydration. Our study highlights the need for additional comprehensive research to assess exposure to multiple risk factors including heat, hydration, and pesticides exposures and a need for them to be considered simultaneously. Currently, most research studies focus on heat-related exposures and kidney function, but there is a paucity of epidemiological studies on pesticide exposure in combination with heat/hydration. Most studies, including this one, that do include these factors based on self-reported exposures, lack specific information on pesticides, and do not include environmental measurements of pesticide or biomarkers (Scammell et al., 2019).

### 4.4. Strengths and limitations

Though there are many strengths of our research, a main strength is that our study is the first to incorporate two groups (organic and conventional) of field workers who were not only working within the same grape farm but working in distinct sections on the farm. This study design has allowed us to better control for potential pesticide exposure among MSFWs assigned to specific types of farming and reduces the variability of other factors that could affect their kidney functioning such as food type variability. For example, the MSFWs working on this specific farm in Sonora typically consumed similar meals to each other and drank from the same water source, since the food and water were provided to all workers by the farm owner. Also, the dorms where MSFWs stayed were relatively like each other, which eliminates some household variability, but increases the possibility for take home pathways and cross contamination between MSFW groups since there was no division of dorms between MSFW who worked in the conventional and organic field areas. Additional research should be done to further reduce cross contamination to truly assess the differences in exposure and health outcomes for MSFWs assigned to conventional and organic fields.

Ultimately, this is a study of many firsts, including the first to be conducted in a migratory farmworker population, the first longitudinal study to be conducted in this region and the first longitudinal study to evaluate the kidney functioning and compare them to heat strain by collecting individual measurements to estimate PSI and dehydration. Our results shows the need of long-term monitoring of farm workers’ kidney function and their occupational exposures, as all MSFWs arrived at the farm with a normal kidney function, but many experienced high risk of kidney injuries and one worker developed kidney disease during the time they worked in this farm. Hence, early detection of kidney injury or disease and prolong surveillance is important in agricultural workers because kidney injuries have been associated with an increased risk of permanent kidney damage, which puts agricultural workers at a higher risk of developing CKD (Bellomo et al., 2004; Moyce et al., 2016). Our study calls for occupational surveillance of agricultural workers in other regions of the world to improve detection and prevent further health issues. Additionally, our findings indicate that not one risk factor in isolation, but a multiple-risk factors are involved in the development of kidney damage in agricultural workers; therefore, future studies should focus on the combination of occupational factors.

Despite the significant components of this work, there are limitations. One of these limitations is that our study lacks the inclusion of other urinary markers for kidney functioning that could have helped to validate our findings. For example, the amount of protein in urine as an indicator of kidney filtration, or the use of other novel urinary biomarkers such as neutrophil gelatinase-associated lipocalin (NGAL), or measuring of serum cystatin C can serve as a renal function marker to detect early acute injury and provide a better understanding of the kidney function in this population (Nadkarni et al., 2017). Also, creatine phosphokinase (CPK) was not analyzed in this study, which has been shown to correlate with potential development of rhabdomyolysis and the risk of AKI (Bieber and Jefferson, 2019). An important limitation is the number of participants that were lost to follow up for the last measurement with an attrition rate of 36% across all participants. Also, there is the possibility for a healthy worker selection effects (HWSE), as the office workers who dropped out from our study were generally older with higher BMI and blood pressure than the office workers who completed the study. Although the attrition rate was relatively high, there were no significant differences in the demographic characteristics and measurements collected from MSFWs who dropped out before the late-harvest and the ones who stayed for the whole study. Another limitation is the use of job categories and insecticide application as a proxy to pesticide exposure, as the true concentration of pesticides and the specific type of pesticides that participants were exposed to is unknown. Direct monitoring of pesticide biomarkers could help improve this study.

One limitation more is that even though PSI may be appropriate to evaluate levels of heat strain in different environments, it may not be the most reliable tool to identify participants who are at a high risk of reaching thermal tolerance limits, as recently discovered by an experimental study with 15 females and 21 males (Davey et al., 2021). However, according to Davey et al. (2021), monitoring the rate of change of the rectal or skin temperature, or heart rate with thermal perceptions could be a valid alternative to predict thermal tolerance limits in extreme environments. Also, although when calculating PSI, rectal temperature is considered the gold standard measurement for core body temperature, ear canal temperature is a validated alternative that is more practical and less invasive (Amoateng-Adjepong et al., 1999; Heidari et al., 2018; Muir et al., 2001; Smitz et al., 2009). While ear temperature readings could be influenced by surrounding environmental conditions and temperature variations within the ear canal, it has been demonstrated that the digital thermometer device used in this study, Braun Thermoscan Exact Temp, can provide a valid core body temperature estimation during high physical activity in heat (Hamouti et al., 2010; Morán-Navarro et al., 2019; Towey et al., 2017).

In addition, heart rate and core body temperature used to estimate PSI were collected intermittently during the two time points of the MSFW and officer workers’ active work-shift and resting periods. Alternately, PSI could be estimated by collecting continuous real-time core body temperature and heart rate throughout the workday. Additionally, the metabolic work rate collected during the first sampling timepoint might be distorted by MSFWs’ lack of time to acclimatize to the new work environment. Also, although BMI is a widely used tool to identify overweight or obese individuals, we recognized that BMI has its limitations since it has low sensitivity and high specificity to identify adiposity in populations with different ethnic and racial backgrounds; hence, future research is needed on the obesity related cutoffs for this Mexican population (Macias et al., 2014; Motamed et al., 2017).

Another weakness is that our study had no gender distribution, as we only recruited male workers. Hence, the results from this study cannot be generalized to all farm workers, as women can be affected differently by occupational exposures due to known gender differences in agricultural work activities and behaviors (Habib et al., 2014; Jayasekara et al., 2019; Moyce et al., 2018). Another limitation is that the race adjustment factors used in the eGFR calculation were validated in a population from the U.S., which does not represent the population groups in this study, even though the serum creatinine-based equations of CKD-EPI are commonly used worldwide. The current eGFR equation used for this study only considers Latinxs and Native Americans within the same group, but it does not consider Indigenous groups from outside the U.S., which is an important limitation because of the large group of Mexican Indigenous workers in this study. Thus, future studies should consider these groups in developing future epidemiological equations to predict eGFR. However, it is important to mention that we obtained similar results when using only serum creatinine in the analyses, so this limitation of the eGFR equation in relation to race is not likely to significantly affect our findings.

## 5. Conclusion

To our knowledge, this is the first longitudinal study that evaluates several occupational risk factors and kidney function in MSFWs. It is also the first study of its kind, collecting longitudinal data from workers in an organic certified field, a conventional field, and a control group to better understand differences in these exposures. Our findings suggest that those employed in high-risk jobs, such as farm work (regardless of whether it takes place on a conventional or organic field or farm) should be frequently monitored and studies should include additional quantitative multifactorial measures of pesticides, heat strain, and biomarkers of kidney function. Future studies should consider a multifactorial approach to evaluate kidney functioning in agricultural workers. Our results indicated that the prolonged exposures to occupational hazards in grape field workers can experience a significant impair of their renal function in comparison to office workers; therefore, long-term occupational surveillance programs to improve early detection and prevent development of CKDu in agricultural workers in other regions of the world are needed.

## Supplementary Material

Supplementary Material

## Figures and Tables

**Table 1:** Sociodemographic, behavioral, hydration and pesticide exposure characteristics of the study population.

	All participants (n = 151)	MSFWs (n = 101)	Office Workers (n = 50)	p-value
***Sociodemographic & Behavior***				
**Age (years), mean (Min-Max)**	28.5 (18–59)	29.4 (18–59)	26.5 (18–51)	0.052
**Primary Language, n (%)**				
Spanish	112 (74)	67 (66)	45 (90)	**0.001**
Indigenous language	39 (26)	34 (34)	5 (10)	
**State of Origin, n (%)**				
Puebla	57 (37)	55 (55)	2 (4)	**<0.001**
Chiapas	47 (31)	41 (41)	6 (12)	
Sonora	40 (27)	0 (0)	40 (80)	
Other	7 (5)	5 (5)	2 (4)	
**Education, n (%)**				
Middle School or less	93 (62)	86 (85)	7 (14)	**<0.001**
High School and above	58 (38)	15 (15)	43 (86)	
**Monthly Income, n(%)**				
Less than $500	116 (77)	86 (85)	30 (60)	**<0.001**
$500 or above	35 (23)	15 (15)	20 (40)	
**Alcohol, n(%)** ^a^	86 (53)	67 (66)	19 (32)	**<0.001**
**Smoking, n(%)** ^a^	57 (38)	46 (46)	11 (22)	**0.003**
**Pain Medications, n(%)** ^a^				
No used of NSAIDs	131 (86.8)	86 (85.2)	45 (90.0)	0.188
Recent used of NSAIDs	20 (13.2)	15 (14.8)	5 (10.0)	
***Hydration and Pesticide Exposure Practices*** ^a^				
**Daily Water Intake, n (%)**				
≤ 4 Liters	131 (86.8)	89 (88.9)	41 (82.0)	0.095
> 4 Liters	20 (13.2)	11 (11.1)	9 (18.0)	
**Recent Sweetened Drinks (Sodas), n(%)**				
No soda consumption	51 (33.5)	23 (22.7)	28 (56.0)	**<0.001**
Recent soda consumption	100 (66.5)	78 (77.3)	22 (44.0)	
**Number of Sweetened Drinks Consumed, n(%)**				
< 1 beverage a week	63 (41.9)	22 (21.4)	45 (90.0)	
1 beverage per day	61 (40.1)	54 (53.8)	4 (8.0)	**<0.001**
> 1 beverages per day	27 (18.0)	25 (24.8)	1 (2.0)	
**Insecticides in Home/Dorms, n(%)**				
Recent application	29 (19.1)	25 (24.8)	5 (10.0)	**0.027**
No recent application	122 (80.9)	76 (75.2)	45 (90.0)	

a Abbreviations: MSFWs, migrant seasonal farm workers; BMI, body mass index; SD, standard deviation, n, sample size. Statistical test performed: *t*-test for age as continuous and chi-square test for categorical variables.Information collected at pre-harvest. Note: participants were asked about recent consumption of alcohol and smoking; one sweetened beverage refers to one soda in aluminum 12 fluid ounce can.

**Table 2:** Pre-harvest anthropometric, hydration, heat stress, and physiological measurements.

	All participants (N = 151)	MSFWs (n = 101)	Office Workers (n = 50)	p-value
***Anthropometric Measurements***				
Weight (kg), mean (SD)	71.0 (12.5)	70.8 (11.4)	71.3 (14.7)	0.700
Height (m), mean (SD)	1.67 (0.7)	1.63 (0.6)	1.73 (0.7)	**<0.001**
BMI (kg/m^2^), mean (SD)	25.9 (4.1)	26.6 (3.9)	23.7 (4.3)	**<0.001**
Normal: BMI < 25, n (%)	68 (45.0)	35 (35.0)	33 (66.0)	
OW: BMI 25–29.9, n (%)	65 (43.0)	50 (50.0)	15 (30.0)	**<0.001**
Obese: BMI *>* 30, n (%)	18 (12.0)	16 (15.0)	2 (4.0)	
***Blood Pressure (mmHg)***				
Systolic, mean (SD)	115.1 (17.5)	116 (16.4)	113.0 (17.7)	0.290
Diastolic, mean (SD)	73.2 (13.1)	74.3 (13.4)	71.0 (9.6)	0.140
***Work intensity, heat stress and dehydration***				
MWR (W/m^2^), mean (SD)	146 (35.6)	169 (43.1)	133 (29.8)	**<0.001**
WBGT_eff_ (°C), mean (SD)	17.3 (2.9)	17.7 (3.4)	13.7 (1.1)	**<0.001**
PSI, GM (GSD)^a^	2.4 (1.3)	2.6 (1.9)	1.8 (1.2)	**<0.001**
TBW (mL/kg), mean (SD)	39.4 (4.5)	38.8 (3.9)	41.0 (5.2)	**0.002**
S-Osmolality (mOsm/kg), mean (SD)	264.0 (17.4)	262.7 (19.5)	263.7 (11.2)	0.740
Urinary Specific Gravity, n (%)				
Hydrated: SG ≤ 1.010	39 (25.7)	25 (25.0)	13 (26.0)	
Mildly Dehydrated: 1.010 *<* SG ≤ 1.021	37 (24.6)	23 (23.0)	17 (34.0)	0.640
Dehydrated: SG *>* 1.021	75 (49.7)	53 (53.0)	20 (40.0)	
***Kidney Function Biomarker***				
SCr (mg/dL), mean (SD)	0.7 (0.1)	0.7 (0.1)	0.8 (0.2)	0.110
eGFR (mL/min/1.73m^2^), mean (SD)	126 (14.2)	125 (13.0)	127 (16.6)	0.390

Abbreviations: MSFWs, migrant seasonal farm workers; BMI, body mass index; OW, overweight; MWR, metabolic work rate; WBGT_eff_, effictive measure of wet and bulb global temperature (8 hour average); PSI, physiological strain index; TBW, total body water; S-OSM, serum osmolality; eGFR, estimated globular filtration rate; SD, standard deviation; SCr, serum creatinin; n, sample size; GM, geometric mean; GSD, geometric standard deviation. Statistical test performed: *t*-test for continuous and chi-square for categorical variables. Bolded p-values are statistically significant.

a Variable was log-normally transformed, GM and GSD are presented.

**Table 3:** Participants with kidney injury based on RIFLE classification during the late harvest.

	MSFWs Organic (n = 37)	MSFWs Conventional (n = 38)	Office Workers (n = 20)
Risk (↓ >25% GFR), n(%)	4 (11)	8 (21%)	0 (0%)
Injury (↓ >50% GFR), n(%)	0 (0%)	2 (5%)	0 (0%)

**Table 4:** MSFWs working in *conventional field* with eGFR below 90 mL/min/1.73 m^2^.

Participant	Pre-harvest (eGFR)	Late harvest (eGFR)	eGFR % Change
MSFW 1*	120.56	33.22	87.34
MSFW 2	146.44	81.23	65.22
MSFW 3	136.81	88.98	47.83
MSFW 4	115.01	81.23	33.79
MSFW 5	119.43	89.24	30.19
MSFW 6	101.35	71.58	29.77
MSFW 7	114.12	85.31	28.82
MSFW 8	111.74	83.53	28.21

Note: eGFR below 60 mL/min/1.73 m^2^ indicates signs of kidney disease, while eGFR <90 mL/min/1.73 m^2^ may indicate signs of kidney damage.

* MSFWs with signs of kidney disease. All MSFWs with values below 90 mL/min/1.73 m^2^ had less than 45 years of age and only worked in the conventional field (no worker in organic field or office worker had eGFR values below 90 mL/min/1.73 m^2^).

**Table 5:** Physiological measurements during the pre-harvest and late harvest by job categories.

	MSFWs in organic fields			MSFWs in conventional fields			Office workers		
	Pre-harvest	Late harvest	p-value	Pre-harvest	Late harvest	p-value	Pre-harvest	Late harvest	p-value
	(n = 37)	(n = 37)		(n = 38)	(n = 38)		(n = 20)	(n = 20)	
**eGFR (mL/min/1.73m^2^)**									
Mean (SD)	127.5 (12.2)	110.9 (13.6)^b^	**<0.001**	127.4 (12.9)	101.2 (19.4)^b^	**<0.001**	127.4 (16.6)	135.4 (14.4)	0.399
**Serum Creatinine (mg/dL)**									
Mean (SD)	0.7 (0.1)	0.9 (0.2)^b^	**<0.001**	0.7 (0.2)	1.0 (0.3)^b^	**<0.001**	0.8 (0.2)	0.7 (0.1)	0.749
**Serum Uric Acid (mg/dL)**									
Mean (SD)	5.0 (1.4)	5.8 (1.1)	**<0.001**	5.6 (1.4)	6.0 (1.2)	**0.013**	5.5 (1.2)	4.8 (1.2)	0.376
**Blood Urea Nitrogen (mg/dL)**									
Mean (SD)	11.1 (2.8)	14.0 (3.7)	**<0.001**	11.7 (3.3)	12.9 (3.4)	**0.003**	13.8 (2.9)	14.7 (3.8)	0.129
**Serum Osmolality (mOsm/kg)**									
Mean (SD)	263.3 (18.8)	270.8 (2.9)	**0.005**	262.2 (20.3)	271.4 (3.9)	**0.001**	263.7 (11.2)	271.1 (5.6)	0.294
**Systolic Blood Pressure (mmHg)**									
Mean (SD)	115.0 (18.7)	127.0 (21.1)	**<0.001**	117.0 (14.0)	123.0 (13.3)	**0.004**	113.0 (17.7)	105.0 (8.9)	0.556
**Diastolic Blood Pressure (mmHg)**									
Mean (SD)	73.4 (15.7)	76.3 (6.3)	0.170	75.5 (10.9)	73.7 (10.9)	0.394	71.0 (9.6)	70.4 (6.2)	0.125
**Body Mass Index (kg/m^2^)**									
Mean (SD)	26.7 (4.1)	26.1 (3.7)	0.212	26.5 (3.1)	25.6 (3.2)	**0.003**	23.7 (4.3)	21.6 (2.9)	**0.025**
**Total Body Water (mL/kg)**									
Mean (SD)	38.7 (4.3)	38.6 (4.2)	0.359	38.7 (3.6)	37.6 (3.6)	**0.002**	41.0 (5.2)	39.0 (3.0)	**0.023**
**Metabolic Work Rate (Watts/m^2^)**									
Mean (SD)	169.0 (45.5)	327.0 (65.1)	**0.001**	170.0 (41.2)	294.0 (58.8)	**<0.001**	133.0 (29.8)	146.0 (35.6)	0.114
**Physiological Strain Index** ^a^									
Geometric Mean (GSD)	2.6 (1.3)	4.1 (1.2)	**<0.001**	2.7 (1.3)	4.1 (1.2)	**<0.001**	1.82 (1.2)	2.2 (1.2)	0.050

a Geometric mean and geometric standard deviation (GSD) are presented: variable was log-normally transformed; Statistical test performed: paired *t*-test. Bolded p-values are statistically significant.

b eGFR was significantly lower in MSFWs at the conventional field than at the organic field during the late harvest, *t*-test (p < 0.001). Abbreviations: MSFWs, migrant seasonal farm workers; eGFR, estimated globular filtration rate; SD, standard deviation, n, sample size.

**Table 6:** Linear mixed effect models evaluating change in eGFR among all participants.

Models	Estimate (β)	SE	(95% CI)
^a.^ **Model 1**			
*Time*			
Early Harvest	*Reference*	-	-
Late Harvest	−16.98	1.65	(−20.22, −13.69)***
*Educational Level*			
Some high school and above	*Reference*	-	-
Middle school or less	−3.00	2.10	(−7.01, 1.16)
*Age & Anthropometric Measurements*			
Body Mass Index^d^	−0.46	0.26	(−0.98, −0.28)*
Participant Age	−0.69	0.11	(−0.91, −0.47)***
Systolic Blood Pressure^d^	−0.10	0.05	(−0.20, 0.01)
^b.^ **Model 2**			
Physiological Strain Index^1^	−0.16	0.03	(−0.15, −0.06)***
*Water Intake* ^d^			
Daily Water Intake (≤4 L)	*Reference*	-	-
Daily Water Intake (>4 L)	−0.43	2.1	(−7.34, 0.29)
*Dehydration - Specific Gravity* ^d^			
Hydrated: SG ≤ 1.010	*Reference*	-	-
Mildly Dehydrated: 1.010 *<* SG ≤ 1.021	−9.68	2.84	(−13.31, −3.26)**
Dehydrated: SG *>* 1.021	−13.62	2.53	(−16.56, −7.71)***
*Educational Level*			
Some High School and above	*Reference*	-	-
Middle School or less	−1.37	1.85	(−4.01, 4.20)
*Age & Anthropometric Measurements*			
Participant Age	−0.60	0.11	(−0.80, −0.37)***
Body Mass Index^d^	−0.16	0.26	(−0.66, 0.36)
Systolic Blood Pressure^d^	−0.08	0.06	(−0.19, 0.03)
^c.^ **Model 3**			
*Job Categories*			
Office Worker	*Reference*	-	-
MSFWs in organic field	−19.02	4.16	(−27.29, −10.82)***
MSFWs in conventional field	−29.17	4.06	(−37.26, −21.17)***
*Insecticide at home/dorm* ^d^			
No recent application	*Reference*	-	-
Recent application	0.92	1.87	(−2.78, 4.64)
*Age & Anthropometric Measurements*			
Participant Age	−0.04	0.10	(−0.86, −0.44)***
Body Mass Index^d^	−0.51	0.23	(−1.00, −0.02)**
Systolic Blood Pressure^d^	−0.04	0.05	(−0.14, 0.10)

a Model 1: The study timepoints adjusted by demographic and anthropometric measurements.

b Model 2: Heat strain and hydration adjusted by anthropometric measurements;

1 As the PSI was log-transformed prior analysis, the estimate (β) of PSI represents a 1% increase in PSI is associated with an average unit decrease in eGFR.

c Model 3 (M3): Pesticide exposure based on individual job categories adjusted by other covariates; results presented in M3 are change in eGFR during study timepoints are the beta coefficient of the time × job category (full model output results presented in Table S9). Abbreviations: eGFR, estimated globular filtration rate, SG, specific gravity; SE, standard error; CI, confidence interval.

* p-value<0.05;

** p-value<0.01;

*** p-value<0.001.

Note: Water intake and recent pesticide application refers to within the last few days.

d Variables measured at each sampling timepoint.

**Table 7:** Final linear mixed effects model (*Model 4*) evaluating change in eGFR among all participants (Interaction between PSI and job category).

Variables	Estimate (β)	SE	(95% CI)	
**Final Model: Heat Stress and Pesticide Exposure**				
*Job Category × PSI* ^a^				
Office Worker × PSI	*Reference*	-	-	
MSFWs in organic field × PSI	−0.23	0.10	(−0.44, −0.03)	*
MSFWs in conventional field × PSI	−0.37	0.11	(−0.57, −0.16)	**
*Water Intake* ^b^				
Daily Water Intake (≤4 L)	*Reference*	-	-	
Daily Water Intake (>4 L)	−0.20	2.14	(−4.21, 3.35)	
*Dehydration - Specific Gravity* ^b^				
Hydrated: SG ≤ 1.010	*Reference*	-	-	
Mildly Dehydrated: 1.010 *<* SG ≤ 1.021	−8.51	2.77	(−13.98, −3.03)	**
Dehydrated: SG *>* 1.021	−11.99	2.48	(−16.88, −7.10)	***
*Insecticide at home/dorm* ^b^				
No recent application	*Reference*	-	-	
Recent application	−2.00	2.01	(−4.62, 3.35)	
*Age & Anthropometric Measurements*				
Participant Age	−0.55	0.10	(−0.75, −0.34)	
BMI^b^	−0.37	0.25	(−0.86, 0.13)	
Systolic Blood Pressure^b^	−0.08	0.06	(−0.18, 0.03)	

Note: The change in eGFR associated with hydration and heat strain × job category adjusted by other covariates including insecticide application at home/dorm;

a As the PSI was log-transformed prior analysis, the estimate (β) of Job category × PSI represents a 1% increase in PSI in MSFWs in the conventional field or organic field associated with an average unit decrease in eGFR (full model output results presented in Table S10). Abbreviations: eGFR, estimated globular filtration rate, SG, specific gravity; SE, standard error; CI, confidence interval.

* p-value<0.05;

** p-value<0.01;

*** p-value*<*0.001.

Note: Water intake and recent pesticide application refers to within the last few days of surveying participants.

b Variables measured at each sampling timepoint.
