# Supplementary material for: Longitudinal assessment of kidney function in migrant farm workers
Source: Environ Res. Author manuscript; Available in PMC 2021 Nov 10. (PMC8578352; doi:10.1016/j.envres.2021.111686)
Supplement: Supplementary Material [file NIHMS1726740-supplement-Supplementary_Material.pdf]

SUPPLEMENTARY MATERIAL

Table S1. Pre-Harvest Demographic Characteristics Among MSFWs (Dropped out versus Completed)

	Dropped out (n = 26)	Completed Study (n = 75)	p-value*
**Origin**			
Puebla, n (%)	12 (46)	36 (48)	
Chiapas, n (%)	12 (46)	38 (51)	0.240
Other, n (%)	2 (8)	1 (1)	
**Primary Language**			
Spanish only, n(%)	18 (68)	45 (60)	0.242
Indigenous language, n(%)	8 (32)	30 (40)	
**Education**			
Less than or equal to Middle School	22 (85)	60 (80)	
Some High School and Above	4 (15)	15 (20)	0.125
**Monthly Income, n(%)**			
Less than $500	21 (84)	67 (89)	
Greater or equal to $500	5 (16	8 (11)	0.100
**Smoking**	14 (54)	32 (42)	0.147
**Alcohol**	15 (58)	50 (66)	0.621
**Previous farm work activities**	17 (66)	51 (68)	0.509

* Chi-square analyses: no significant differences between participants who stayed versus dropouts

Table S2. Pre-Harvest Physiological Measurements Among MSFWs (Dropped out versus Completed)

	Dropped out (n = 26)	Completed Study (n = 75)	p-value
Age (years)	30.0 (8.5)	28.8 (9.3)	0.480
BMI	27.1 (3.8)	26.3 (3.4)	0.475
Systolic BP (mm Hg)	117.6 (17.7)	115.1 (15.3)	0.220
Diastolic BP (mm Hg)	72.7 (14.3)	75.8 (12.7)	0.228
Physiological Strain Index	2.7 (0.6)	2.7 (0.7)	0.266
Metabolic Work Rate (Watts/m^2)	174.5 (48.5)	166.0 (38.4)	0.279
Serum Osmolality (mOSM/kg)	265.4 (15.8)	260.6 (21.8)	0.411
Serum Uric Acid (mg/dL)	5.3 (1.5)	5.3 (1.3)	0.420
Serum Creatinine (mg/dL)	0.7 (0.1)	0.7 (0.1)	0.447
eGFR (mL/min/1.73 m^2)	125.1 (11.2)	129.2 (13.6)	0.089

*T-test; Mean, SD.

Table S3. Pre-Harvest Measurements among office workers only (Dropped out versus Continued)

	Dropped out (n = 30)	Stayed for full Study (n = 20)	p-value*
Smoking, n (%)	11 (37)	0.0 (0)	-
Age (years)	29.4 (8.4)	22.1 (5.5)	0.010
BMI	25.4 (4.2)	21.0 (2.9)	<0.001
Systolic BP (mm Hg)	119.3 (3.5)	103.6 (10.5)	0.001
Diastolic BP (mm Hg)	72.7 (10.3)	68.5 (8.1)	0.128
Physiological Strain Index	1.8 (0.2)	1.9 (0.4)	0.119
Metabolic Work Rate (Watts/m^2)	134.6 (26.8)	130.8 (34.3)	0.285
Serum Osmolality (mOSM/kg)	259.5 (12.5)	169.9 (3.8)	0.001
Serum Uric Acid (mg/dL)	5.9 (1.1)	5.0 (1.0)	0.030
Serum Creatinine (mg/dL)	0.8 (0.1)	0.7 (0.2)	0.060
eGFR (mL/min/1.73 m^2)	120.9 (12.5)	127.1 (17.7)	0.050

*Wilcoxon Sum rank Test; Mean, SD.

Table S4. Pre-Harvest Measurements of MSFWs in Conventional versus Organic Field

	Organic Area (n = 50)	Conventional Area (n = 51)	p-value
BMI	26.7 (4.1)	26.5 (3.1)	0.718
Systolic BP (mm Hg)	115 (18.7)	117 (14.0)	0.509
Diastolic BP (mm Hg)	73.4 (15.7)	75.5 (10.9)	0.401
Heart Rate (resting)	66.5 (8.8)	65.3 (8.3)	0.358
Heart Rate (working)	73.7 (10.5)	75.5 (10.4)	0.466
Ear Temperature (resting)	36.4 (0.4)	36.5 (0.5)	0.678
Ear Temperature (working)	37.8 (0.3)	37.9 (0.4)	0.559
Physiological Strain Index	2.6 (1.3)	2.7 (1.3)	0.457
Metabolic Work Rate (Watts/m^2)	169 (45.5)	170 (41.1)	0.888
Serum Osmolality (mOSM/kg)	263.3 (18.8)	262.2 (20.3)	0.756
Serum Uric Acid (mg/dL)	5.0 (1.4)	5.6 (1.4)	0.051
Serum Creatinine (mg/dL)	0.7 (0.1)	0.7 (0.2)	0.568
eGFR (mL/min/1.73 m^2)	127.5 (12.2)	127.9 (12.4)	0.168

No significant differences between MSFWs in conventional versus organic field (based on t-test)

Table S5. Pre-Harvest Characteristics of MSFWs

	Organic Area (n = 50)	Conventional Area (n = 51)	p-value
**Age, mean (range)**	29.7 (18 - 48)	30.6 (18 - 59)	0.827
**Primary Language**			
Spanish only, n(%)	36 (72)	31 (61)	
Indigenous language, n(%)	14 (28)	20 (39)	0.213
**Origin, n (%)**			
Puebla, n (%)	33 (66)	22 (43)	
Chiapas, n (%)	16 (32)	25 (49)	
Other, n (%)	1 (2)	4 (8)	0.125
**Education, n (%)**			
Less than or equal to Middle School	46 (92)	40 (78)	
Some High School and Above	4 (8)	11 (22)	0.259
**Monthly Income, n (%)**			
Less than $500	43(86)	43 (84)	0.859
Greater or equal to $500	7 (14)	8 (16)	
**Smoking, n (%)**	24 (48)	22 (43)	0.519
**Alcohol, n (%)**	33 (66)	34 (67)	0.519
**Recent NSAIDs Usage, n (%)**	7 (14)	8 (15)	0.333
**Previous farm work activities, n (%)**	36 (73)	32 (62)	0.193
**Experienced working in grape farming (%)**			
More than a year of experience	18 (34)	17 (33)	
First time in grape farming	32 (66)	34 (67)	0.571
**Recent insecticide app in home/dorm, n (%)**	12 (26)	13 (25)	0.229

Note: There were no significant difference between MSFWs in conventional versus organic field (based on chi-square test).

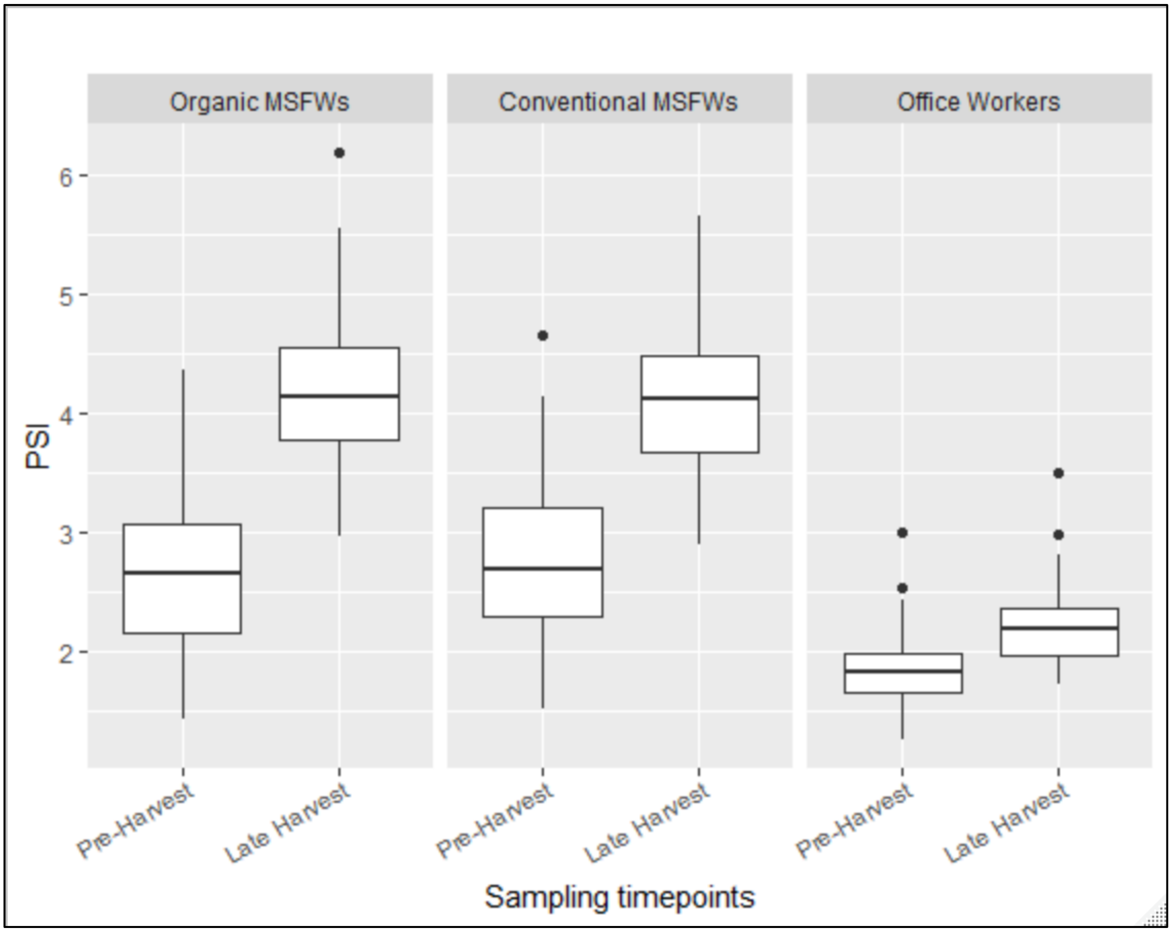

Figure S1. Changes in PSI during study timepoints by job category.

As presented in Table S6, the time of sampling was an important factor, as participants during the late harvest (July) had a mean eGFR that was 17.79 mL/min/1.73m^2, significantly lower than at the pre-harvest (March) ($p < 0.001$). Education level was significantly associated with eGFR, as participants who reported receiving less than or equal to a middle school education had an average of 9.33 mL/min/1.73m^2 lower eGFR than participants that reported receiving some high school education ($p < 0.001$). Other demographic characteristics, such as primary language and monthly income, were significantly associated ($p < 0.2$) with eGFR, as participants who reported an Indigenous language as their primary language along with participants who made less than $500 per month had lower eGFR. All of the anthropometric measurements, including participants' age, BMI, and systolic blood pressure, were significantly associated with a decrease in eGFR of 0.81, 1.10, and 0.33 mL/min/1.73m^2, respectively. Diastolic blood pressure was not associated with participants' eGFR. The 8-hr average $WBGT_{eff}$ and the metabolic work rate were significantly associated with a 1.57 and 0.09 mL/min/1.73m^2 decrease in eGFR, respectively ($p < 0.001$). The PSI was log-normally transformed before analyses. There was a significant association with eGFR. A 1% increase in participants' PSI is associated with eGFR decline of 0.24 mL/min/1.73m^2 ($p < 0.001$) (Table S6).

Also presented in Table S6, job categories were significantly associated with eGFR, as MSFWs working in the organic field and MSFWs working in the conventional field had significant decreases in eGFR of 10.50 and 15.53 mL/min/1.73m^2, respectively, compared to office workers ($p < 0.001$). Participants' recent reporting of the application of insecticides inside their homes or dormitories was significantly associated with a decrease in eGFR of 6.43 mL/min/1.73m^{2}in comparison to participants who did not apply insecticides in their home/dormitories ($p = 0.01$) (Table S6). In terms of dehydration measured with specific gravity (SG), participants who had experienced mild dehydration or dehydration had significantly lower eGFR (11.52 and 18.14 mL/min/1.73m^2, respectively) compared to hydrated participants ($p < 0.001$) (Table S6). Additionally, participants with a daily water consumption of more than 4 liters during their workday had a significant decrease in eGFR of 10.36 mL/min/1.73m^2 compared to participants with a daily water intake less than or equal to 4 liters ($p<0.001$). Participants who reported recent consumption of soda had a mean eGFR that was 23.33 mL/min/1.73m^2, which was significantly lower than participants without recent soda consumption ($p = 0.01$) (Table S6). Alcohol consumption, use of NSAIDs, and smoking among participants were not significantly associated with eGFR during the study (Table S6). Other subclinical early kidney injury biomarkers in serum were significantly associated with the decline of eGFR in participants. For every unit increase of BUN in participants' serum, their eGFR decreased 1.24 mL/min/1.73m^2 (95% CI: -1.68, -0.79); also, for every unit increase of uric acid in serum, a decline of 5.45 (95% CI: -6.78, -4.11) of eGFR was observed during the study (Table S7).

Table S6. Simple linear mixed effect regression analysis for the associations of eGFR and each independent variable experienced by all participants at work during the study

	Estimate (β)	SE	p-value	
Time				
Pre-Harvest	*Reference*	-	-	
Late Harvest	-17.79	1.58	<0.001	***
Primary Language				
Spanish	*Reference*	-	-	
Indigenous language	-3.91	2.19	0.080	
**Experienced working in grape farming**				
More than a year of experience	*Reference*	-	-	
First time working in grape farming	0.69	1.04	0.514	
Education				
Some High School and Above	*Reference*	-	-	
Less than and equal to Middle School	-9.33	1.98	<0.001	***
Alcohol and Smoking$^{\Psi}$				
No Recent Alcohol Consumption	*Reference*	-	-	
Recent Alcohol Consumption	4.52	3.32	0.380	
No Recent Smoking	*Reference*	-	-	
Recent Smoking	-0.91	2.02	0.650	
Pain Medications$^{\Psi}$				
Not recent used of NSAIDs	*Reference*	-	-	
Recent used of NSAIDs	-2.84	2.60	0.280	
Age & Anthropometric Measurements				
Participant Age	-0.81	0.11	<0.001	***
BMI (kg/m^2), mean (SD) $^{\Psi}$	-1.10	0.28	<0.001	***
Systolic blood pressure $^{\Psi}$	-0.33	0.07	<0.001	***
Diastolic blood pressure $^{\Psi}$	-0.17	0.10	0.30	
Heat Stress & Work Intensity $^{\Psi}$				
8-hr average $WBGT_{eff}$ (˚C)	-1.57	0.10	<0.001	***
ln_PSI	-0.24	2.66	<0.001	***
Metabolic work rate (Watts/m^2)	-0.11	0.01	<0.001	***
Hydration and Behavior$^{\Psi}$				
Hydrated: SG ≤ 1.010	*Reference*	-	-	
Mildly Dehydrated: 1.010 < SG ≤ 1.021	-11.52	2.77	<0.001	***
Dehydrated: SG > 1.021	-18.14	2.37	<0.001	***
Daily Water Intake$^{\Psi}$				
Daily Water Intake (≤ 4 Liters)	*Reference*	-	-	
Daily Water Intake (> 4 Liters)	-10.36	2.17	<0.001	***
Recent Sweetened Drinks (Sodas) $^{\Psi}$				
Not a recent soda consumption	*Reference*	-	-	
Recent soda consumption	-23.33	9.33	0.010	**
Job Categories				
Office Worker	*Reference*	-	-	
MSFWs in organic field	-10.50	2.75	<0.001	***
MSFWs in conventional field	-15.53	2.71	<0.001	***
Insecticides in Home/Dorms$^{\Psi}$				
No recent application	*Reference*	-	-	

Yes, recent application	-6.43	2.42	0.010	**

** p-value<0.01; *** p-value<0.001; ᴪ Measurement taken at each sampling timepoint; *Recent* refers last two days

Table S7. Simple linear mixed effect regression analysis for the associations of eGFR and early kidney injury markers.

	Estimate (β)	SE	p-value	
**Serum Uric Acid (mg/dL)**	-5.45	0.68	<0.001	***
**Osmolality (mOsm/kg)**	-0.41	0.06	<0.001	***
**Blood Urea Nitrogen (mg/dL)**	-*1.24*	*0.22*	<0.001	***

*** p-value<0.001.

Table S8a. The calculated variance inflation factor (VIF) utilized to verify multicollinearity among variables of interest (Type of Worker).

	VIF	df
Time	1.12	1
Age	1.13	1
BMI	1.33	1
Education	1.81	1
Systolic Pressure	1.20	1
Job Category	2.01	2
Insecticide at home/dorm	1.11	1

Note: Collinearity existed between Education and type of worker (Education was removed from final analysis).

Table S8b. The calculated variance inflation factor (VIF) utilized to verify multicollinearity among variables of interest (Type of Worker).

	VIF	df
Time	1.12	1
Age	1.11	1
BMI	1.31	1
Systolic Pressure	1.20	1
Job Category	1.28	2
Insecticide at home/dorm	1.11	1

Note: Removed education

Table S8c. The calculated variance inflation factor (VIF) utilized to verify multicollinearity among variables of interest (PSI).

	VIF	df
Time	2.64	1
Age	1.18	1
BMI	1.40	1
Systolic Pressure	1.26	1
Education	1.30	1
Specific Gravity	1.26	2
Metabolic work rate	4.65	1
Ln_PSI	3.56	1
Water Intake	1.18	1

Note: VIF > 2.0 - collinearity between PSI, metabolic work rate and time.

Table S8d. The calculated variance inflation factor (VIF) utilized to verify multicollinearity among variables of interest (PSI).

	VIF	df
Age	1.18	1
BMI	1.27	1
Systolic Pressure	1.24	1
Education	1.29	1
Specific Gravity	1.22	2
Ln_PSI	1.34	1
Water Intake	1.14	1

In a post-hoc analysis, we examined the multicollinearity between our primary variables and the selected significant variables from the simple linear mixed effect analyses including time, education, metabolic work rate, hydration, heat stress, age and BMI (Table S6). The variables for time, metabolic work rate and $WBGT_{eff}$ were excluded from the second model, due to the high VIF and correlation coefficients between $WBGT_{eff}$, time, and metabolic work rate (Table S8a-d, Figure S3.1).

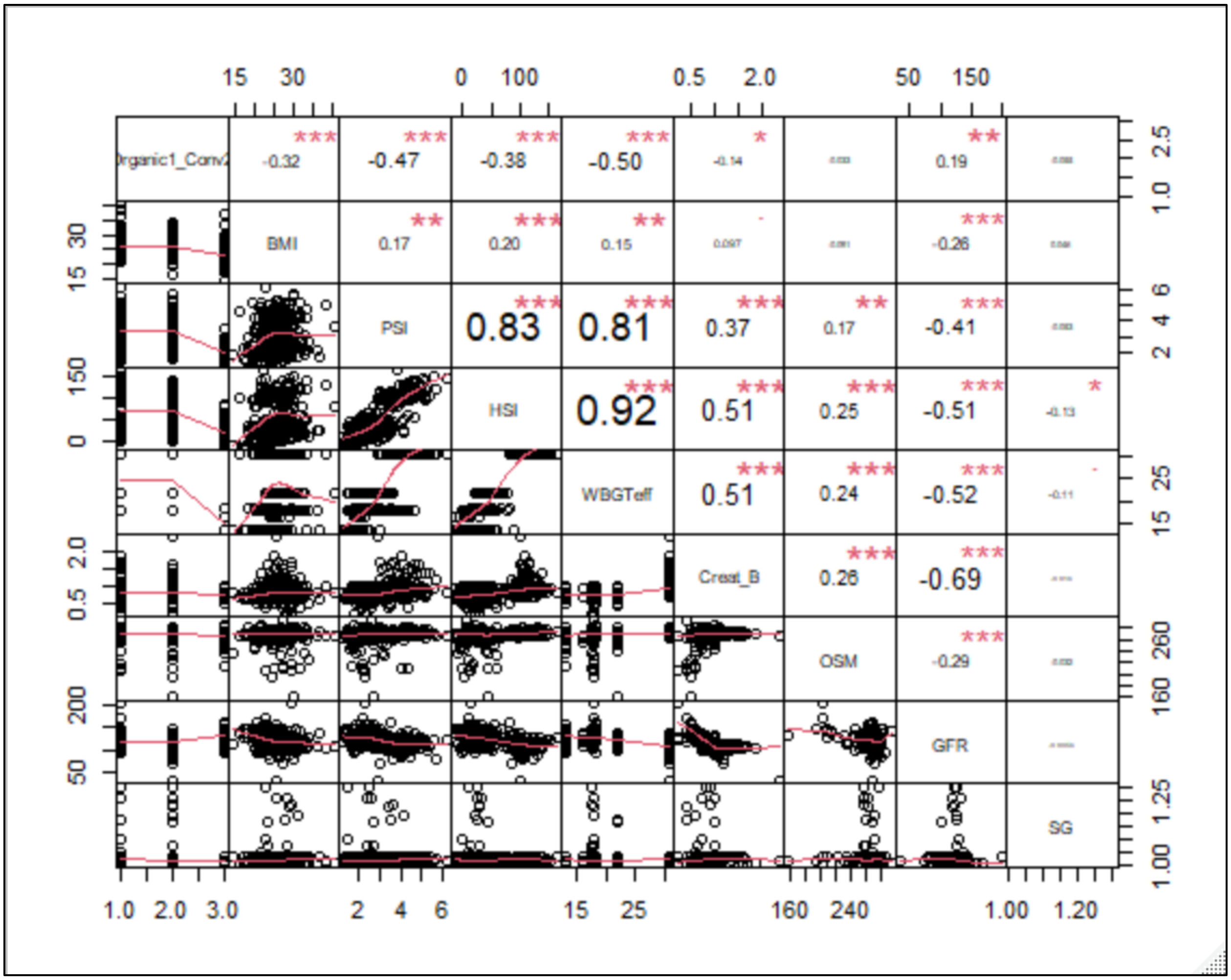

Figure S2. Correlation coefficients used to verify multicollinearity. Note: WBGT and HSI were not included because of collinearity with PSI. * p<0.05; ** p-value<0.01; ***p-value<0.001

After the univariate regression analyses shown in table S6 and after testing for multicollinearity, the variables included in Model 1 includes: time, educational level, BMI, age, and systolic blood pressure (SBP); Model 2: PSI, water intake, education, age, BMI, and SBP; Model 3: Job type x time, insecticide application in dorms, age, BMI, and SBP; Model 4: Job Category x PSI, water intake and specific gravity, insecticide application in dorms, age, BMI, and SBP.

Table S9. Linear mixed effects model (model 3) evaluating change in eGFR by job category (Pesticide Exposure – job category x Time)

	Estimate (β)	SE	95% CI	
(Intercept)	161.80	7.41	(147.22, 176.25)	***
Time				
Pre-Harvest	*Reference*	-	-	
Late Harvest	2.62	3.38	(-4.05, 9.38)	
Job Category				
Office Worker	*Reference*	-	-	
Organic Worker	3.49	2.77	(-1.16, 8.81)	
Conventional Worker	3.38	2.74	(-2.04, 8.77)	
Job Category x Time				
Office Worker x Pre-Harvest	*Reference*	-	-	
Organic Worker x Pre-Harvest	-19.02	4.16	(-27.29, -10.82)	***
Conventional Worker x Pre-Harvest	-29.17	4.06	(-37.26, -21.17)	***
Insecticide at home/dorm Ψ				
No recent application	*Reference*	-	-	
Recent application	0.92	1.87	(-2.78, 4.64)	
Participant Age	-0.04	0.10	(-0.86, -0.44)	***
BMI Ψ	-0.51	0.23	(-1.00, -0.02)	**
Systolic Blood Pressure Ψ	-0.04	0.05	(-0.14, 0.10)	

* p<0.05; ** p-value<0.01; ***p-value<0.001; Ψ Measurement taken at each sampling timepoint; *Recent* refers to las two days.

Table S10. Final linear mixed effects model (Model 4) evaluating change in eGFR among MSFWs and office workers (Interaction between PSI and job category)

Variables	Estimate (β)	SE	(95% CI)	
**Final Model: Heat Stress and Pesticide Exposure**				
Job Category x PSI ᵠ				
Office Worker x PSI	*Reference*	-	-	
MSFWs in organic field x PSI	-0.23	0.10	(-0.44, -0.03)	*
MSFWs in conventional field x PSI	-0.37	0.10	(-0.57, -0.16)	**
Water Intake ᵠ				
Daily Water Intake (≤ 4 Liters)	*Reference*	-	-	
Daily Water Intake (> 4 Liters)	-0.21	2.14	(-4.21, 3.35)	
Specific Gravity ᵠ				
Hydrated: SG ≤ 1.010	*Reference*	-	-	
Mildly Dehydrated: 1.010 < SG ≤ 1.021	-8.51	2.77	(-13.98, -3.03)	**
Dehydrated: SG > 1.021	-11.99	2.48	(-16.88, -7.10)	***
Insecticide at home/dorm ᵠ				
No recent application	*Reference*	-	-	
Recent application	-2.00	2.01	(-4.62, 3.35)	
Age & Anthropometric Measurements				
Participant Age	-0.55	0.10	(-0.75, -0.34)	***
BMI ᵠ	-0.37	0.25	(-0.86, 0.13)	
Systolic Blood Pressure ᵠ	-0.08	0.06	(-0.18, 0.03)	

* p<0.05; ** p-value<0.01; ***p-value<0.001. ᵠ Measurement taken at each sampling timepoint; *Recent* refers to las two days. Note: PSI was log-transformed before analyses (β/100 was presented when this variable is involved).

Table S11. Final linear mixed effects model evaluating change in *serum creatinine* among all participants (Interaction between PSI and job category)

Variables	Estimate (β)	SE	(95% CI)	
**Final Model: Heat Stress and Pesticide Exposure**				
Job Category x PSI [1 Ψ]				
Office Worker x PSI	*Reference*	-	-	
MSFWs in organic field x PSI	0.27	0.12	(0.02, 0.52)	*
MSFWs in conventional field x PSI	0.37	0.13	(0.12, 0.62)	***
Water Intake [Ψ]				
Daily Water Intake (≤ 4 Liters)	*Reference*	-	-	
Daily Water Intake (> 4 Liters)	0.02	0.02	(-0.05, 0.05)	
Specific Gravity [Ψ]				
Hydrated: SG ≤ 1.010	*Reference*	-	-	
Mildly Dehydrated: 1.010 < SG ≤ 1.021	0.10	0.03	(0.02, 0.15)	**
Dehydrated: SG > 1.021	0.14	0.03	(0.08, 0.20)	***
Insecticide at home/dorm [Ψ]				
No recent application	*Reference*	-	-	
Recent application	0.04	0.03	(-0.05, 0.04)	
Age & Anthropometric Measurements				
Participant Age [a]	0.02	0.01	(-0.05, 0.03)	
BMI [a Ψ]	0.03	0.02	(-0.03, 0.09)	
Systolic Blood Pressure [a Ψ]	0.08	0.04	(-0.01, 0.10)	

[a.] For every 10 units increase in age, BMI, or SBP. A t-test evaluating the differences between the serum creatinine levels of MSFWs showed that the workers in the organic area had a significantly lower creatinine compared to workers in the conventional area during the late harvest (p=0.008); [Ψ] Measurement taken at each sampling timepoint; *Recent* refers to las two days. * p-value<0.05; ** p-value<0.01; ***p-value<0.001

Table S12. Sensitivity analysis of the final linear mixed effects model (model 4): comparation a reduced model and an imputed model using MICE to evaluate change in eGFR among all participants

Variables (outcome eGFR)	Reduced Model (n = 95)				MICE Model (n = 151)			
Job Category x PSI [1]	**Estimate (β)**	**SE**	**(95% CI)**		**Estimate (β)**	**SE**	**(95% CI)**	
Office Worker x PSI	*Reference*	-	-		*Reference*	-	-	
MSFWs in organic field x PSI	-0.22	0.13	(-0.47, -0.03)	*	-0.23	0.10	(-0.44, -0.03)	*
MSFWs in conventional field x PSI	-0.27	0.12	(-0.51, -0.02)	**	-0.37	0.11	(-0.57, -0.16)	**
Water Intake								
Daily Water Intake (≤ 4 Liters)	*Reference*	-	-		*Reference*	-	-	
Daily Water Intake (> 4 Liters)	-0.19	3.41	(-6.96, 6.50)		-0.21	2.14	(-4.21, 3.35)	
Specific Gravity								
Hydrated: SG ≤ 1.010	*Reference*	-	-		*Reference*	-	-	
Mildly Dehydrated: 1.010 < SG ≤ 1.021	-5.39	3.73	(-12.76, 1.98)		-8.51	2.77	(-13.98, -3.03)	**
Dehydrated: SG > 1.021	-13.36	3.42	(-20.12, -6.56)	***	-11.99	2.48	(-16.88, -7.10)	***
Insecticide at home/dorm								
No recent application	*Reference*	-	-		*Reference*	-	-	
Recent application	-2.46	2.62	(-7.62, 2.72)		-2.00	2.01	(-4.62, 3.35)	
Age & Anthropometric Measurements								
Participant Age	-0.66	0.14	(-0.94, -0.38)	***	-0.55	0.1	(-0.75, -0.34)	***
BMI	-0.19	0.25	(-0.89, 0.52)		-0.37	0.25	(-0.86, 0.13)	
Systolic Blood Pressure	-0.06	0.07	(-0.20, 0.09)		-0.08	0.06	(-0.18, 0.03)	
R^2		0.464				0.498		

Reduced Model refers to the model used after deleting all missing values & only ID's with completed repeated measures were kept
MICE Model (Multiple Imputation Chain Equation): analyses used in this study to evaluate eGFR in MSFWs and office workers for missing data; Ψ Measurement taken at each sampling timepoint; Recent refers to las two days. * p-value<0.05; ** p-value<0.01; ***p-value<0.001

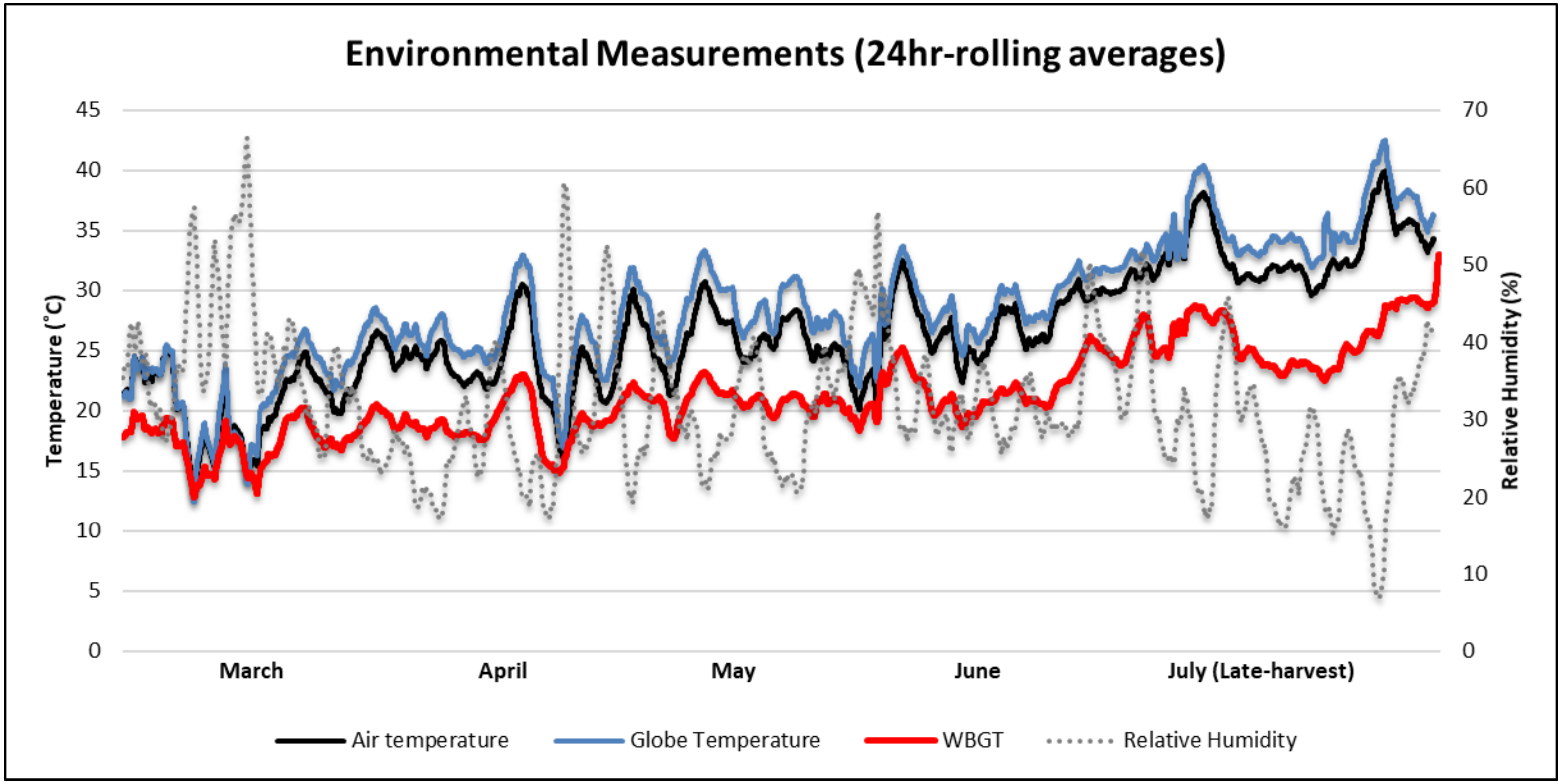

Figure S3 Some environmental measures taken every minute (24-hr rolling averages) for the length of the study.

Table S13. Linear mixed effect models evaluating change in eGFR among MSFWs only

Models	Estimate (β)	SE	(95% CI)	
[a.] **Model 1**				
Time				
Early Harvest	*Reference*	-	-	
Late Harvest	-21.65	1.88	(-25.36, -17.93)	***
Educational Level				
Some high school and above	*Reference*	-	-	
Less than and equal to middle school	-1.90	2.68	(-7.20, 3.39)	
Age & Anthropometric Measurements				
Body Mass Index$^{\Psi}$	-0.40	0.30	(-0.99, 0.20)	
Participant Age	-0.49	0.12	(-0.72, -0.27)	***
Systolic Blood Pressure$^{\Psi}$	-0.03	0.06	(-0.15, 0.10)	
[b.] **Model 2**				
Physiological Strain Index [1]	-0.24	0.04	(-0.31, -0.16)	***
Water Intake $^{\Psi}$				
Daily Water Intake (≤ 4 Liters)	*Reference*	-	-	
Daily Water Intake (> 4 Liters)	0.55	2.49	(-4.35, 5.44)	
Dehydration - Specific Gravity $^{\Psi}$				
Hydrated: SG ≤ 1.010	*Reference*	-	-	
Mildly Dehydrated: 1.010 < SG ≤ 1.021	-5.68	3.84	(-13.23, 1.87)	
Dehydrated: SG > 1.021	-8.18	3.27	(-14.62, -1.75)	*
Educational Level				
Some High School and Above	*Reference*	-	-	
Less than and equal to Middle School	-4.37	2.79	(-9.85, 1.12)	
Age & Anthropometric Measurements				
Participant Age	-0.42	0.12	(-0.66, -0.18)	***
Body Mass Index $^{\Psi}$	-0.24	0.32	(-0.87, 0.39)	
Systolic Blood Pressure $^{\Psi}$	-0.10	0.07	(-0.22, 0.04)	
[c.] **Model 3**				
Job Categories				
MSFWs in organic field	*Reference*	-	-	
MSFWs in conventional field	-10.15	3.44	(-16.96, -3.35)	**
Insecticide at home/dorm $^{\Psi}$				
No recent application	*Reference*	-	-	
Recent application	0.22	2.04	(-3.84, 4.28)	
Age & Anthropometric Measurements				
Participant Age	-0.49	0.11	(-0.71, -0.27)	***
Body Mass Index $^{\Psi}$	-0.49	0.30	(-1.07, 0.10)	
Systolic Blood Pressure $^{\Psi}$	-0.04	0.05	(-0.16, 0.07)	

[a.] Model 1: The study timepoints adjusted by demographic and anthropometric measurements. [b.]Model 2: Heat strain and hydration adjusted by anthropometric measurements; [1]As the PSI was log-transformed prior analysis, the estimate (β) of PSI represents a 1% increase in PSI is associated with an average unit decrease in eGFR. [c] Model 3 (M3): Pesticide exposure based on individual job categories adjusted by other covariates; results presented in M3 are change in eGFR during study timepoints are the beta coefficient of the time x job category. Abbreviations: eGFR, estimated globular filtration rate, SG, specific gravity; SE, standard error; CI, confidence interval. * p-value<0.05; ** p-value<0.01; ***p-value<0.001. Note: Water intake and recent pesticide application refers to within the last few days. $^{\Psi}$ Variables measured at each sampling timepoint.

Table S14. Final linear mixed effects model evaluating change in eGFR among MSFWs only (Interaction between PSI and job category)

Variables	Estimate (β)	SE	(95% CI)	
**Final Model: Heat Stress and Pesticide Exposure**				
Job Category x PSI [1]				
MSFWs in organic field x PSI	*Reference*	-	-	
MSFWs in conventional field x PSI	-0.14	0.06	(-0.27, -0.02)	*
Water Intake ᵠ				
Daily Water Intake (≤ 4 Liters)	*Reference*	-	-	
Daily Water Intake (> 4 Liters)	0.41	2.47	(-4.45, 5.27)	
Dehydration - Specific Gravity ᵠ				
Hydrated: SG ≤ 1.010	*Reference*	-	-	
Mildly Dehydrated: 1.010 < SG ≤ 1.021	-5.66	3.79	(-13.12, 1.80)	
Dehydrated: SG > 1.021	-7.92	3.22	(-14.26, -1.58)	*
Insecticide at home/dorm ᵠ				
No recent application	*Reference*	-	-	
Recent application	-1.30	2.26	(-5.75, 3.14)	
Age & Anthropometric Measurements				
Participant Age	-0.43	0.12	(-0.66, -0.19)	***
BMI ᵠ	-0.29	0.32	(-0.91, 0.33)	
Systolic Blood Pressure ᵠ	-0.11	0.07	(-0.24, 0.02)	

Note: The change in eGFR associated with hydration and heat strain x job category adjusted by other covariates including insecticide application at home/dorm; [1] As the PSI was log-transformed prior analysis, the estimate (β) of Job category x PSI represents a 1% increase in PSI in MSFWs in the conventional field associated with an average unit decrease in eGFR. Abbreviations: eGFR, estimated globular filtration rate, SG, specific gravity; SE, standard error; CI, confidence interval. * p-value<0.05; ** p-value<0.01; ***p-value<0.001. Note: Water intake and recent pesticide application refers to within the last few days of surveying participants. ᵠ Variables measured at each sampling timepoint.
